# Machine learning-guided determination of *Acinetobacter* density in waterbodies receiving municipal and hospital wastewater effluents

**DOI:** 10.1038/s41598-023-34963-6

**Published:** 2023-05-12

**Authors:** Temitope C. Ekundayo, Mary A. Adewoyin, Oluwatosin A. Ijabadeniyi, Etinosa O. Igbinosa, Anthony I. Okoh

**Affiliations:** 1 SAMRC Microbial Water Quality Monitoring Centre, University of Fort Hare, Alice, Eastern Cape South Africa; 2 Department of Biotechnology and Food Science, Durban University of Technology, Steve Biko Campus, Steve Biko Rd, Musgrave, Berea, 4001 Durban South Africa; 3 Department of Microbiology, University of Medical Sciences Ondo, Ondo, Nigeria; 4 Department of Biological Sciences, Faculty of Natural, Applied and Health Sciences, Anchor University, Ayobo Road, Ipaja, P. M. B. 001, Lagos, Nigeria; 5 Department of Microbiology, Faculty of Life Sciences, University of Benin, Private Mail Bag 1154, Benin City, 300283 Nigeria; 6 Department of Environmental Health Sciences, College of Health Sciences, University of Sharjah, P.O. Box 27272, Sharjah, United Arab Emirates

**Keywords:** Environmental microbiology, Policy and public health in microbiology, Microbiology, Applied microbiology

## Abstract

A smart artificial intelligent system (SAIS) for *Acinetobacter* density (AD) enumeration in waterbodies represents an invaluable strategy for avoidance of repetitive, laborious, and time-consuming routines associated with its determination. This study aimed to predict AD in waterbodies using machine learning (ML). AD and physicochemical variables (PVs) data from three rivers monitored via standard protocols in a year-long study were fitted to 18 ML algorithms. The models’ performance was assayed using regression metrics. The average pH, EC, TDS, salinity, temperature, TSS, TBS, DO, BOD, and AD was 7.76 ± 0.02, 218.66 ± 4.76 µS/cm, 110.53 ± 2.36 mg/L, 0.10 ± 0.00 PSU, 17.29 ± 0.21 °C, 80.17 ± 5.09 mg/L, 87.51 ± 5.41 NTU, 8.82 ± 0.04 mg/L, 4.00 ± 0.10 mg/L, and 3.19 ± 0.03 log CFU/100 mL respectively. While the contributions of PVs differed in values, AD predicted value by XGB [3.1792 (1.1040–4.5828)] and Cubist [3.1736 (1.1012–4.5300)] outshined other algorithms. Also, XGB (MSE = 0.0059, RMSE = 0.0770; R^2^ = 0.9912; MAD = 0.0440) and Cubist (MSE = 0.0117, RMSE = 0.1081, R^2^ = 0.9827; MAD = 0.0437) ranked first and second respectively, in predicting AD. Temperature was the most important feature in predicting AD and ranked first by 10/18 ML-algorithms accounting for 43.00–83.30% mean dropout RMSE loss after 1000 permutations. The two models' partial dependence and residual diagnostics sensitivity revealed their efficient AD prognosticating accuracies in waterbodies. In conclusion, a fully developed XGB/Cubist/XGB-Cubist ensemble/web SAIS app for AD monitoring in waterbodies could be deployed to shorten turnaround time in deciding microbiological quality of waterbodies for irrigation and other purposes.

## Introduction

*Acinetobacter* species belong to the group of aerobic gram-negative bacteria that are non-motile, non-fermentative, catalase positive, oxidase negative encapsulated coccobacilli, having a DNA G+C content of 39 to 47 mol^1,2^. Taxonomically, scientists have identified 68 validated species in the genus *Acinetobacter*, with numerous others yet to be delineated into species^3–5^. Many *Acinetobacter* species are found naturally in different environments, including soil, water, air, wastewater, fomites, human skin, animals, and even on plants^6–8^. Some species can utilise different substrates, such as amino acids, carbohydrates, organic acids, and hydrocarbons, while some can secrete industrial enzymes like lipase and protease^9,10^. However, few species are human opportunistic pathogens. For instance, *Acinetobacter baumannii* is a well-known notorious species in hospital settings that cause life-threatening infections such as pneumonia, respiratory and urinary tract infections, septicaemia, and wound infections, among others, especially in immune-compromised patients^11–13^.

*Acinetobacter* species are widely spread via the environmental milieu and may alarmingly spread antimicrobial resistance genes in the environment^14,15^. In addition, wastewater treatment plants (WWTPs) feed by hospital and municipal wastewater inflows have been reported to contribute multidrug-resistant (MDR), and extensively drug-resistant (XDR) *Acinetobacter* isolates to their effluents receiving waterbodies compared with other sources^15,16^. Discharging WWTP effluents increases the prevalence of *Acinetobacter* in the receiving river waterbodies and promotes antimicrobial resistance and transmission to irrigated vegetables^15^. The transmission of *Acinetobacter* spp. (especially *A. baumannii*)—with high antimicrobial resistance and case fatality ratio—onto fresh produce has been demonstrated and reviewed by Carvalheira et al.^17^. *Acinetobacter* species with different resistant capabilities ranging from MDR to XDR have been isolated in fresh fruits and vegetables (apples, cabbages, melons, cauliflowers, peppers, mushrooms, lettuce, cucumbers, bananas, radishes, sweet corn carrots, potatoes, peach, pear, strawberry, apple, celery, tomato, and radish) at a density up to 50–1000 CFU/g^18^ in Hong Kong^19^, France^20^, Nigeria^21^, Lebanon^22^, Portugal^23^ and agricultural environment in Algeria^24^. Furthermore, waterbodies especially rural rivers for instance, support recreational use of considerably high levels by people incognizant of the inflow/inputs of WWTP effluents and the influx of multidrug-resistant pathogens of public health concern including *Acinetobacter*^25^.

The routine experimental determination and identification of *Acinetobacter* species and other bacteria in all matrices (water, food, and clinical samples, etc.) using most probable number, direct plate count, adenosine triphosphate testing, and membrane filtration methods are usually laborious, repetitive, time-consuming (incubation period), and cost-intensive endeavours that required expert knowledge which might not be readily available in most settings. Therefore, there is an urgent need for rapid, reliable, and cost-effective means that required no or low technical know-how to assess *Acinetobacter* density (AD) in waterbodies and other matrices to ensure short turnaround time necessary to make informed microbiological quality decisions. It is hypothesized that AD in waterbodies could be predicted accurately and dependably by using machine learning intelligence frameworks that depend upon the dynamic’s relationship between AD based on the afore determination methods and physicochemical variables of waterbody and other matrices in a low-cost and time-effective way. Thus, an artificial intelligence system for AD determination in waterbodies receiving WWTP effluents, which are subsequently used as irrigation source waters (ISW), would be an invaluable preventive option for immediate and future public health challenges.

The main merits of ML models lie in their capacity to overcome problems associated with traditional statistical models in capturing and predicting multidimensional interactions in large data by “learning” deep patterns^26^. ML frameworks and SAIS allow proactive management of events rather than reactive. Thus, MLs and SAIS are finding increasing applications in many sectors, including medicine, precision farming, environmental management, water purification, *Vibrio* abundance on microplastics, wastewater treatment, watershed typologies and stormwater quality and epidemiology prediction^26–30^ and the application is endlessly expanding daily.

Therefore, the present study aimed at predicting/determining AD in waterbodies (receiving hospital, municipal and WWTP effluents) using ML without the repetitive, laborious, cost-intensive, and time-consuming laboratory routines to reduce the turnaround time essential to make informed microbiological quality decisions (e.g., for irrigation use and other purposes).

## Materials and methods

### Sample collection and in-situ determination of physicochemical data

Water samples were collected using grab sampling technique from the Great Fish River, Keiskamma River and Thyume River, serving as receiving waterbodies for municipal and hospital wastewater effluents (MHWE) discharge at one or more points along their courses in the Eastern Cape Province, South Africa. At least, five strategic sampling locations based on socioeconomic importance (e.g., fishing, swimming, nearness to wastewater treatment plants, farming, pasture, irrigation, dam etc.) of each river were selected for sample collection. At the sampling sites, water temperature (TEMP), pH, total dissolved solids (TDS), electrical conductivity (EC), salinity (SAL), and dissolved oxygen (DO) were determined in-situ using a standard multi-parameter device (Hanna, model HI 9828) instrumental protocol. In addition, the rivers’ turbidity (TBS) was assessed using a turbidimeter (HACH, model 2100P). For microbiological analysis and biochemical oxygen demand (BOD) measurement, midstream water samples (25–30 cm depth) were collected at the same sampling sites in three replicates into sterile glass and amber bottles, respectively and stored in iceboxes and transported to the laboratory for analysis with 6 h of collection^31^. After five days of incubation of samples in amber bottles, the BOD of the samples was determined using a biochemical oxygen demand meter (HACH, HQ 40 days)^31^. Detailed sampling strategy, sampling points’ description, and study area maps were as described in our previous study^32^.

### *Acinetobacter* data acquisition

The density of *Acinetobacter* species in the water samples was estimated via membrane filtration^31^. Briefly, 100 ml of serially diluted water samples were filtered in three independent iterations using a Ø47 mm 0.45 μm pore-sized cellulose membrane^31^. These membranes were aseptically placed onto freshly prepared *Acinetobacter* CHROMagar plates containing selective supplements (CHROMagar, Paris, France) per the manufacturer’s instruction. The plates were incubated at 37 °C for 24 h. All *Acinetobacter* colonies presented as red colouration on CHROMagar plates post-incubation was counted and log transformed (log CFU/100 mL). All isolates were purified, validated as oxidase negative, and assessed by *Acinetobacter*-specific polymerase chain reaction. Fifty per cent (50%) of glycerol stocks of the pure culture was prepared and stored at – 80 °C.

### Model development

#### Pre-processing and modelling procedure

The datasets were first subjected to explanatory and bivariate Pearson's correlation (r) [Eq. (1)] analyses. The estimation of 95% confidence intervals (95% CI) of the r-value in bivariate correlation analysis was based on Fisher's r-to-z transformation with bias adjustment [Eq. (2)]. To avoid multicollinearity, where the r-value between two variables ≥ 0.99, one of them was dropped randomly in subsequent models (see Table 2). Any of the two variables can be used in the implementation of the models. Also, for models’ implementation, the datasets were centre scaled such that the mean = 0 and the square root of the variance = 1 for variables. The dataset for DTR was not scaled.

1 $$r=\sum_{i=1}^{h}({u}_{i}-\overline{u })({w}_{i}-\overline{w })/\sqrt{\sum_{i=1}^{h}{({u}_{i}-\overline{u })}^{2}}\sqrt{\sum_{i=1}^{n}{{(w}_{i}-\overline{w })}^{2}} $$

2 $$z=\mathrm{arctanh}\left(r\right)= 1/2\mathrm{log}((1+r)/(1-r))$$

where r is a Pearson’s correlation coefficient with possible values from − 1 to 1 inclusive. Here, u and w represent a pair of PVs and h is the sample size.

*Acinetobacter* density (AD) was modelled as a dependent variable of the rivers’ physicochemical variables (PVs). Hence, the conditional expected (CE) AD value at instances of PVs consisting of a vector of TEMP, DO, BOD, TSS, SAL, and pH is derived as ${\mathrm{CE}}_{AD|PVs}(AD)$. Thus, the estimation of the mean AD can be constructed as Eq. (3).

3 $${\mathrm{CE}}_{AD|PVs}(AD)\approx f\left(PVs\right).$$

Equation (1) was implemented via 18 regression ML algorithms that have the robust capability to fit multidimensional variables of ordinal/continuous outcome, including linear regression with stepwise selection (LRSS), an RF, XGB, SVR, linear regression (LR), a gradient boosted machine (GBM), neural network (NNT) (6–6–1 network with 49 weights multiple; decay = 0.1), a KNN (k-nearest neighbour), M5P, a boosted regression tree (BRT), a Cubist regression, a decision tree (DTR), multivariate adaptive regression splines (MARS), ANN [with one 6-node hidden layers (ANN6), extreme learning machine (ELM), two 4- and 2- node hidden layers (ANN42), and two 3- and 3-node hidden layers (ANN33), and elastic net (ENR)]. The dataset (540 observations, 6 variables after explanatory feature selection) was split into a learning subset (70%) for the estimate of models’ coefficients and a validation subset (30%) for model substantiation. In all the ML implementations of Eq. (1), ten different learning-validation dataset pairs were generated via tenfold cross-validation accompanied by 3 repeats and 10 tune-lengths. Optimal hyper-parameters were derived and selected through a grid search algorithm. Models’ hyper-parameters are provided in detail in the supplemental material. Detailed discussion on the strengths and weaknesses and previous application of the various algorithms could be found elsewhere and their documentation.

The explanatory rendition of all variables contributions in the models was according to Eq. (4):

4 $$f\left(w.\right)= {t}_{0} +\sum_{j=1}^{p}t\left(j,w.\right),$$

where *t*(*j, w.)* denotes the jth variable contribution measure to the model’s prediction at instance *w* and *t*_*0*_ is the average model prediction^33^.

### Assessment of ML model’s performance

The MLI algorithms model’s performance was determined against experimental data based on Eqs. (5)–(8):

5 $$\mathrm{Mean \,  squared}-\mathrm{error}: MSE\left(f,\underline{U}, \underline{w}\right)=\frac{1}{h}\sum_{i}^{h}{\left({\widehat{w}}_{i}-{w}_{i}\right)}^{2}= \frac{1}{h}\sum_{1}^{h}{r}_{i}^{2}$$

6 $$\mathrm{Root}-\mathrm{mean}-\mathrm{squared  \, error}: RMSE\left(f,\underline{U}, \underline{w}\right)= \sqrt{MSE\left(f,\underline{U}, \underline{w}\right)}$$

7 $${R}^{2}\left(f,\underline{U}, \underline{w}\right)=1- \frac{MSE\left(f,\underline{U}, \underline{w}\right)}{MSE\left({f}_{0},\underline{U}, \underline{w}\right)}$$

8 $$\mathrm{Median  \, absolute  \, deviation}: MAD\left(f,U, \underline{w}\right)=median\left(\left|{r}_{1}\right|, \ldots , \left|{r}_{n}\right|\right).$$

where *h* = number of the sample; f_0_(): baseline model; r_i_: residual for the i_th_ observation, U: matrix of PVs; $\underline{w}$: vector of AD; $f\left(\widehat{\underline{\theta }},\underline{U}\right):$ model based on the training dataset; $\widehat{\underline{\theta }}:$ estimated values of the model’s coefficients; and ${\widehat{\underline{w}}}_{i}:$ model’s prediction equivalent to ${\mathrm{w}}_{i}$.

RMSE was further employed in assessing mean dropout loss for variable importance following 1000 permutation^34,35^.

### Models’ sensitivity analysis

Residual diagnostics and partial-dependence profiles of PVs on the predicted AD was generated to assess the model’s sensitivity. The partial-dependence profile of a model *f*() (i.e., anticipated/predicted AD value at an instance by the model) and the outcome variable *U*^*j*^ set at *s* (over the empirical/marginal distribution of *U*^*-j*^ (h), i.e., the collective distribution of all other PVs without *U*^*j*^ ) is created according to Eqs. (9) and (10):

9 $${q}_{P}^{j}\left(\mathrm{s}\right)={E}_{{\underline{X}}^{-j}}\left\{f\left({U}^{j|=s}\right)\right\}.$$

10 $${\widehat{q}}_{P}^{j}\left(\mathrm{s}\right)=\frac{1}{h}\sum_{i=1}^{h}f\left({\underline{u}}_{i}^{j|=s}\right).$$

The implementation of all models was achieved in R v.4.1.2 software.

## Results

A descriptive summary of the physicochemical variables and *Acinetobacter* density of the waterbodies is presented in Table 1. The mean pH, EC, TDS, and SAL of the waterbodies was 7.76 ± 0.02, 218.66 ± 4.76 µS/cm, 110.53 ± 2.36 mg/L, and 0.10 ± 0.00 PSU, respectively. While the average TEMP, TSS, TBS, and DO of the rivers was 17.29 ± 0.21 °C, 80.17 ± 5.09 mg/L, 87.51 ± 5.41 NTU, and 8.82 ± 0.04 mg/L, respectively, the corresponding DO5, BOD, and AD was 4.82 ± 0.11 mg/L, 4.00 ± 0.10 mg/L, and 3.19 ± 0.03 log CFU/100 mL respectively.

**Table 1:** Descriptive statistics of the physicochemical variables and *Acinetobacter* density of the waterbodies.

Variable	Mean ± SE (min–max)
pH	7.76 ± 0.02 (5.05–9.11)
EC (µS/cm)	218.66 ± 4.76 (47.00–561.00)
TDS (mg/L)	110.53 ± 2.36 (23.00–279.00)
SAL (PSU)	0.10 ± 0.00 (0.02–0.27)
TEMP (°C)	17.29 ± 0.21 (4.74–28.64)
TSS (mg/L)	80.17 ± 5.09 (1.00–1244.00)
TBS (NTU)	87.51 ± 5.41 (4.00–1312.00)
DO (mg/L)	8.82 ± 0.04 (6.66–11.27)
DO5 (mg/L)	4.82 ± 0.11 (0.21–9.72)
BOD (mg/L)	4.00 ± 0.10 (0.52–10.19)
*Acinetobacter* (log CFU/100 mL)	3.19 ± 0.03 (1.00–4.56)

The bivariate correlation between paired PVs varied significantly from very weak to perfect/very strong positive or negative correlation (Table 2). In the same manner, the correlation between various PVs and AD varies. For instance, negligible but positive very weak correlation exist between AD and pH (r = 0.03, p = 0.422), and SAL (r = 0.06, p = 0.184) as well as very weak inverse (negative) correlation between AD and TDS (r = − 0.05, p = 0.243) and EC (r = − 0.04, p = 0.339). A significantly positive but weak correlation occurs between AD and BOD (r = 0.26, p = 4.21E−10), and TSS (r = 0.26, p = 1.09E−09), and TBS (r = 0.26, 1.71E-09) whereas, AD had a weak inverse correlation with DO5 (r = − 0.39, p = 1.31E−21). While there was a moderate positive correlation between TEMP and AD (r = 0.43, p = 3.19E−26), a moderate but inverse correlation occurred between AD and DO (r = − 0.46, 1.26E−29).

**Table 2:** Bivariate correlational relationship among physicochemical variables and *Acinetobacter* density in waterbodies receiving municipal and hospital wastewater effluents.

S/n	Bivariate affinity	r-value (95% CI^a^)	p-value	S/n	Bivariate affinity	r-value (95% CI^a^)	p-value
1	pH vs EC	0.24 (0.16–0.32)	9.72E−09	30	SAL vs TBS	0.14 (0.06–0.22)	0.001
2	pH vs TDS	0.24 (0.16–0.31)	2.6E−08	31	SAL vs DO	0.15 (0.06–0.23)	0.001
3	pH vs SAL	0.24 (0.16–0.32)	1.94E−08	32	SAL vs DO5	− 0.32 (− 0.40 to − 0.24)	1.61E−14
4	pH vs TEMP	0.22 (0.13–0.30)	3.9E−07	33	SAL vs BOD	0.43 (0.36–0.50)	1.03E−25
5	pH vs TSS	0.13 (0.05–0.22)	0.002	34	SAL vs AD	− 0.06 (− 0.14–0.03)	0.184
6	pH vs TBS	0.13 (0.05–0.21)	0.002	35	TEMP vs TSS	0.28 (0.20–0.35)	6.02E−11
7	pH vs DO	− 0.17 (− 0.25 to − 0.09)	5.05E−05	36	TEMP vs TBS	0.28 (0.20–0.35)	6.43E−11
8	pH vs DO5	− 0.19 (− 0.27 to − 0.10)	1.15E−05	37	TEMP vs DO	− 0.80 (− 0.83 to − 0.77)	8.4E−123
9	pH vs BOD	0.14 (0.06–0.23)	0.001	38	TEMP vs DO5	− 0.58 (− 0.63 to − 0.52)	1.13E−49
10	pH vs AD	0.03 (− 0.0–0.12)	0.422	39	TEMP vs BOD	0.34 (0.26–0.41)	1.19E−15
11	EC vs TDS	0.99 (0.99–0.99)	0	40	TEMP vs AD	0.43 (0.36–0.50)	3.19E−26
12	EC vs SAL	1.00 (1.00–1.00)	0	41	TSS vs TBS	1.00 (1.00–1.00)	0
13	EC vs TEMP	− 0.07 (− 0.1–50.01)	0.097	42	TSS vs DO	− 0.38 (− 0.45 to − 0.30)	8.77E−20
14	EC vs TSS	0.14 (0.06–0.22)	0.001	43	TSS vs DO5	− 0.21 (− 0.29 to − 0.13)	1.07E−06
15	EC vs TBS	0.14 (0.06–0.23)	0.001	44	TSS vs BOD	0.08 (0.00–0.17)	0.052
16	EC vs DO	0.13 (0.04–0.21)	0.003	45	TSS vs AD	0.26 (0.18–0.34)	1.09E−09
17	EC vs DO5	− 0.33 (− 0.4 to − 0.26)	1.89E−15	46	TBS vs DO	− 0.38 (− 0.45 to − 0.30)	7.49E−20
18	EC vs BOD	0.43 (0.36–0.50)	3.35E−26	47	TBS vs DO5	− 0.20 (− 0.28 to − 0.12)	1.93E−06
19	EC vs AD	− 0.04 (− 0.13–0.04)	0.339	48	TBS vs BOD	0.08 (− 0.01–0.16)	0.071
20	TDS–SAL	0.99 (0.98–0.99)	0	49	TBS vs AD	0.26 (0.17–0.33)	1.71E−09
21	TDS–TEMP	− 0.05 (− 0.13–0.04)	0.267	50	DO vs DO5	0.52 (0.45–0.57)	4.9E−38
22	TDS vs TSS	0.14 (0.06–0.22)	0.001	51	DO vs BOD	− 0.18 (− 0.26 to − 0.10)	2.19E−05
23	TDS vs TBS	0.14 (0.06–0.22)	0.001	52	DO vs AD	− 0.46 (− 0.52–-0.39)	1.26E−29
24	TDS vs DO	0.10 (0.02–0.19)	0.016	53	DO5 vs BOD	− 0.94 (− 0.95 to − 0.92)	2.3E−246
25	TDS vs DO5	− 0.35 (− 0.42 to − 0.28)	3.22E−17	54	DO5 vs AD	− 0.39 (− 0.46 to − 0.32)	1.31E−21
26	TDS vs BOD	0.45 (0.38–0.51)	7.19E−28	55	BOD vs AD	0.26 (0.18–0.34)	4.21E−10
27	TDS vs AD	− 0.05 (− 0.13–0.03)	0.243	30	SAL vs TBS	0.14 (0.06–0.22)	0.001
28	SAL vs TEMP	− 0.10 (− 0.18–-0.01)	0.026	31	SAL vs DO	0.15 (0.06–0.23)	0.001
29	SAL vs TSS	0.14 (0.05–0.22)	0.001				

a. Estimation is based on Fisher's r-to-z transformation with bias adjustment.

### Model predicted AD and explanatory contribution of PVs

**Figure 1:**
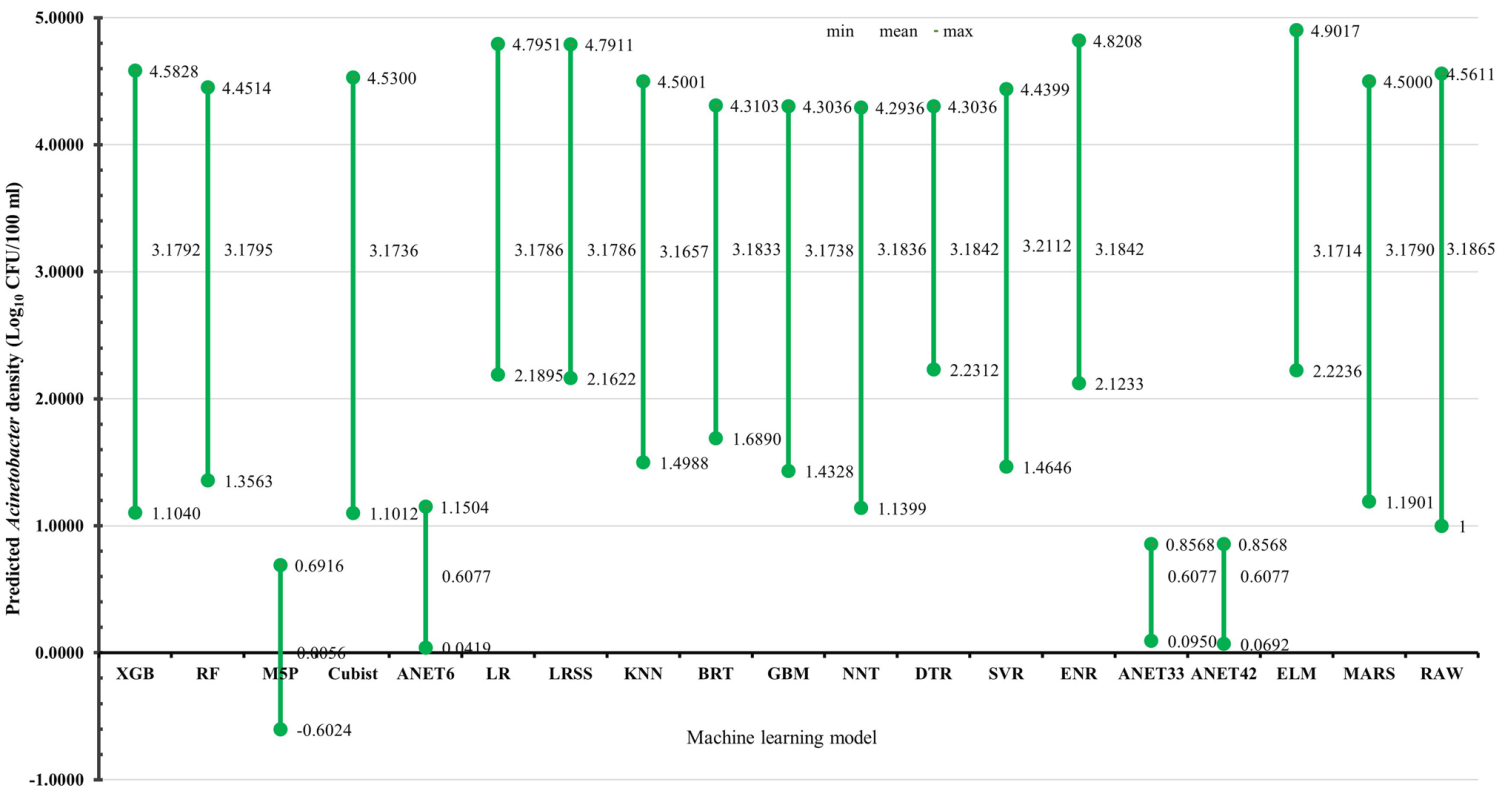
Comparison of ML model-predicted AD in the waterbodies. *RAW* raw/empirical AD value.

The predicted AD by the 18 ML regression models varied both in average value and coverage (range) as shown in Fig. 1. The average predicted AD ranged from 0.0056 log units by M5P to 3.2112 log unit by SVR. The average AD prediction declined from SVR [3.2112 (1.4646–4.4399)], DTR [3.1842 (2.2312–4.3036)], ENR [3.1842 (2.1233–4.8208)], NNT [3.1836 (1.1399–4.2936)], BRT [3.1833 (1.6890–4.3103)], RF [3.1795 (1.3563–4.4514)], XGB [3.1792 (1.1040–4.5828)], MARS [3.1790 (1.1901–4.5000)], LR [3.1786 (2.1895–4.7951)], LRSS [3.1786 (2.1622–4.7911)], GBM [3.1738 (1.4328–4.3036)], Cubist [3.1736 (1.1012–4.5300)], ELM [3.1714 (2.2236–4.9017)], KNN [3.1657 (1.4988–4.5001)], ANET6 [0.6077 (0.0419–1.1504)], ANET33 [0.6077 (0.0950–0.8568)], ANET42 [0.6077 (0.0692–0.8568)], and M5P [0.0056 (− 0.6024–0.6916)]. However, in term of range coverage XGB [3.1792 (1.1040–4.5828)] and Cubist [3.1736 (1.1012–4.5300)] outshined other models because those models overestimated and underestimated AD at lower and higher values respectively when compared with raw data [3.1865 (1–4.5611)].

**Figure 2:**
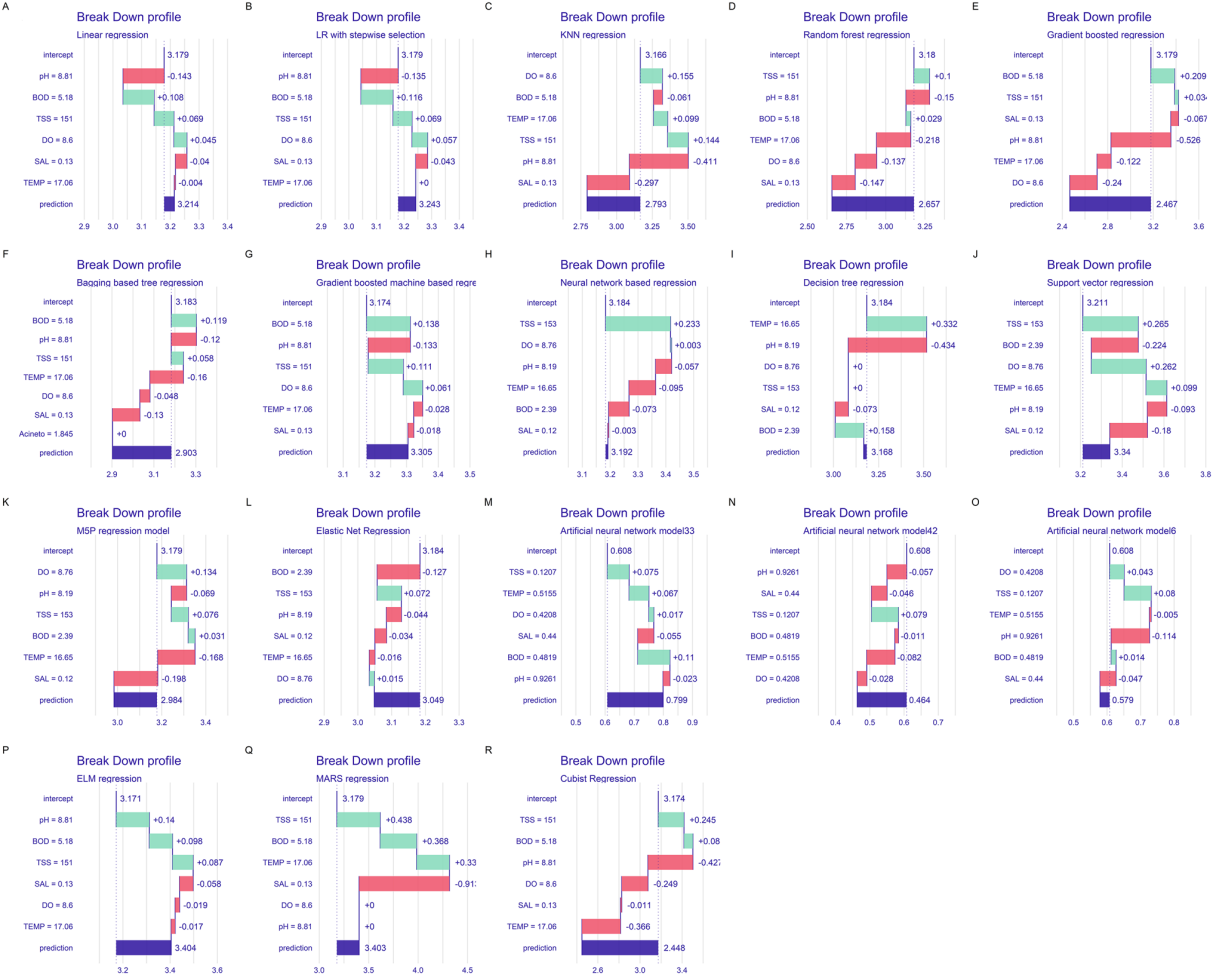
PV-specific contribution to eighteen ML models forecasting capability of AD in MHWE receiving waterbodies. The average baseline value of PV in the ML is presented on the y-axis. The green/red bars represent the absolute value of each PV contribution in predicting AD.

Figure 2 represents the explanatory contributions of PVs to AD prediction by the models. The subplot A-R gives the absolute magnitude (representing parameter importance) by which a PV instance changes AD prediction by each model from its mean value presented in the vertical axis. In LR, an absolute change from the mean value of pH, BOD, TSS, DO, SAL, and TEMP corresponded to an absolute change of 0.143, 0.108, 0.069, 0.0045, 0.04, and 0.004 units in the LR’s AD prediction response/value. Also, an absolute response flux of 0.135, 0.116, 0.069, 0.057, 0.043, and 0.0001 in AD prediction value was attributed to pH, BOD, TSS, DO. SAL, and TEMP changes, respectively, by LRSS. Similarly, absolute change in DO, BOD, TEMP, TSS, pH, and SAL would achieve 0.155, 0.061. 0.099, 0.144, and 0.297 AD prediction response changes by KNN. In addition, the most contributed or important PV whose change largely influenced AD prediction response was TEMP (decreases or decreases the responses up to 0.218) in RF. Summarily, AD prediction response changes were highest and most significantly influenced by BOD (0.209), pH (0.332), TSS (0.265), TEMP (0.6), TSS (0.233), SAL (0.198), BOD (0.127), BOD (0.11), DO (0.028), pH (0.114), pH (0.14), SAL(0.91), and pH (0.427) in XGB, BTR, NNT, DTR, SVR, M5P, ENR, ANET33, ANNET64, ANNET6, ELM, MARS, and Cubist, respectively.

Table 4 presents the eighteen regression algorithms’ performance predicting AD given the waterbodies PVs. In terms of MSE, RMSE, and R^2^, XGB (MSE = 0.0059, RMSE = 0.0770; R^2^ = 0.9912) and Cubist (MSE = 0.0117, RMSE = 0.1081, R^2^ = 0.9827) ranked first and second respectively, to outmatched other models in predicting AD. While MSE and RMSE metrics ranked ANET6 (MSE = 0.0172, RMSE = 0.1310), ANRT42 (MSE = 0.0220, RMSE = 0.1483), ANET33 (MSE = 0.0253, RMSE = 0.1590), M5P (MSE = 0.0275, RMSE = 0.1657), and RF (MSE = 0.0282, RMSE = 0.1679) in the 3, 4, 5, 6, and 7 position among the MLs in predicting AD, M5P (R^2^ = 0.9589 and RF (R^2^ = 0.9584) recorded better performance in term of R-squared metric and ANET6 (MAD = 0.0856) and M5P (MAD = 0.0863) in term of MAD metric among the 5 models. But Cubist (MAD = 0.0437) XGB (MAD = 0.0440) in term of MAD metric.

The feature importance of each PV over permutational resampling on the predictive capability of the ML models in predicting AD in the waterbodies is presented in Table 3 and Fig. S1. The identified important variables ranked differently from one model to another, with temperature ranking in the first position by 10/18 of the models. In the 10 algorithms/models, the temperature was responsible for the highest mean RMSE dropout loss, with temperature in RF, XGB, Cubist, BRT, and NNT accounting for 0.4222 (45.90%), 0.4588 (43.00%), 0.5294 (50.82%), 0.3044 (44.87%), and 0.2424 (68.77%) respectively, while 0.1143 (82.31%),0.1384 (83.30%), 0.1059 (57.00%), 0.4656 (50.58%), and 0.2682 (57.58%) RMSE dropout loss was attributed to temperature in ANET42, ANET10, ELM, M5P, and DTR respectively. Temperature also ranked second in 2/18 models, including ANET33 (0.0559, 45.86%) and GBM (0.0793, 21.84%). BOD was another important variable in forecasting AD in the waterbodies and ranked first in 3/18 and second in 8/18 models. While BOD ranked as the first important variable in AD prediction in MARS (0.9343, 182.96%), LR (0.0584, 27.42%), and GBM (0.0812, 22.35%), it ranked second in KNN (0.2660, 42.69%), XGB (0.4119, 38.60); BRT (0.2206, 32.51%), ELM (0.0430, 23.17%), SVR (0.1869, 35.77%), DTR (0.1636, 35.13%), ENR (0.0469, 21.84%) and LRSS (0.0669, 31.65%). SAL rank first in 2/18 (KNN: 0.2799; ANET33: 0.0633) and second in 3/18 (Cubist: 0.3795; ANET42: 0.0946; ANET10: 0.1359) of the models. DO ranked first in 2/18 (ENR [0.0562; 26.19%] and LRSS [0.0899; 42.51%]) and second in 3/18 (RF [0.3240, 35.23%], M5P [0.3704, 40.23%], LR [0.0584, 27.41%]) of the models.

**Table 3:** Feature importance of PVs over 100 permutational resampling on AD prediction.

Rank	KNN			RF			XGB			SVR			M5P			MARS		
	PE	MDt_loss	%MDt loss	PE	MDt_loss	%MDt loss	PE	MDt_loss	%MDt loss	PE	MDt_loss	%MDt loss	PE	MDt_loss	%MDt loss	PE	MDt_loss	%MDt loss
0	Baseline	0.6231	0	Baseline	0.9198	0	Baseline	1.0670	0	Baseline	0.5226	0	Baseline	0.9206	0	TSS	1.1912	233.28
1	SAL	0.2799	44.92	TEMP	0.4222	45.90	TEMP	0.4588	43.00	DO	0.2094	40.06	TEMP	0.4656	50.58	BOD	0.9343	182.96
2	BOD	0.2660	42.69	DO	0.3240	35.23	BOD	0.4119	38.60	BOD	0.1869	35.77	DO	0.3704	40.23	Baseline	0.5107	100.00
3	TEMP	0.2645	42.45	BOD	0.3169	34.46	DO	0.3853	36.11	TEMP	0.1665	31.87	BOD	0.3241	35.20	SAL	0.5062	99.14
4	DO	0.2532	40.64	TSS	0.2254	24.51	SAL	0.3124	29.27	TSS	0.1403	26.85	SAL	0.2180	23.68	TEMP	0.4839	94.76
5	pH	0.1818	29.18	SAL	0.2034	22.11	TSS	0.2911	27.28	pH	0.1249	23.91	pH	0.1673	18.17	DO	0.2181	42.72
6	TSS	0.1528	24.53	pH	0.1572	17.10	pH	0.2159	20.24	SAL	0.1240	23.73	TSS	0.1516	16.46	pH	0.0000	0.00

Rank	Cubist			BRT			NNT			DTR			ENR			ANET33		
	PE	MDt_loss	%MDt loss	PE	MDt_loss	%MDt loss	PE	MDt loss	%MD loss	PE	MDt_loss	%MDt loss	PE	MDt_loss	%MDt loss	PE	MDt_loss	%MDt loss
0	Baseline	1.0418	0	Baseline	0.6785	0	Baseline	0.3525	0	Baseline	0.4657	0	Baseline	0.2147	0	Baseline	0.1218	0
1	TEMP	0.5294	50.82	TEMP	0.3044	44.87	TEMP	0.2424	68.77	TEMP	0.2682	57.58	DO	0.0562	26.19	SAL	0.0633	51.94
2	SAL	0.3795	36.43	BOD	0.2206	32.51	TSS	0.1284	36.42	BOD	0.1636	35.13	BOD	0.0469	21.84	TEMP	0.0559	45.86
3	BOD	0.3262	31.31	DO	0.1931	28.47	BOD	0.0736	20.88	pH	0.1101	23.64	SAL	0.0160	7.45	TSS	0.0529	43.43
4	DO	0.3118	29.93	TSS	0.1259	18.56	pH	0.0532	15.09	DO	0.0866	18.60	TSS	0.0146	6.80	DO	0.0424	34.82
5	TSS	0.2779	26.68	SAL	0.1072	15.80	DO	0.0354	10.04	TSS	0.0409	8.78	TEMP	0.0135	6.29	BOD	0.0418	34.29
6	pH	0.2190	21.02	pH	0.0799	11.77	SAL	0.0010	0.29	SAL	0.0252	5.40	pH	0.0035	1.65	pH	0.0128	10.47

Rank	ANET42			ANET10			ELM			LR			LRSS			GBM		
	PE	MDt_loss	%MDt loss	PE	MDt_loss	%MDt loss	PE	MDt_loss	%MDt loss	PE	MDt_loss	%MDt loss	PE	MDt_loss	%MDt loss	PE	MDt_loss	%MDt loss
0	Baseline	0.1389	0	Baseline	0.1662	0	Baseline	0.1858	0	Baseline	0.2129	0	Baseline	0.2115	0	Baseline	0.3633	0
1	TEMP	0.1143	82.31	TEMP	0.1384	83.30	TEMP	0.1059	57.00	BOD	0.0584	27.42	DO	0.0899	42.51	BOD	0.0812	22.35
2	SAL	0.0946	68.13	SAL	0.1359	81.76	BOD	0.0430	23.17	DO	0.0584	27.41	BOD	0.0669	31.65	TEMP	0.0793	21.84
3	BOD	0.0903	65.00	DO	0.1021	61.45	TSS	0.0344	18.52	TSS	0.0233	10.93	TSS	0.0233	11.01	TSS	0.0510	14.05
4	pH	0.0567	40.82	BOD	0.0680	40.94	SAL	0.0227	12.23	SAL	0.0101	4.75	SAL	0.0115	5.45	DO	0.0491	13.51
5	TSS	0.0381	27.41	pH	0.0559	33.66	DO	0.0025	1.32	TEMP	0.0058	2.73	pH	0.0045	2.11	SAL	0.0160	4.41
6	DO	0.0361	26.02	TSS	0.0546	32.84	pH	− 0.0042	− 2.27	pH	0.0051	2.41	TEMP	0.0000	0.00	pH	0.0148	4.07

*MDt_loss* mean_dropout_loss, $$\%MDt loss=\mathrm{MDt}\_\mathrm{loss due to a PV}/baseline.$$

**Figure 3:**
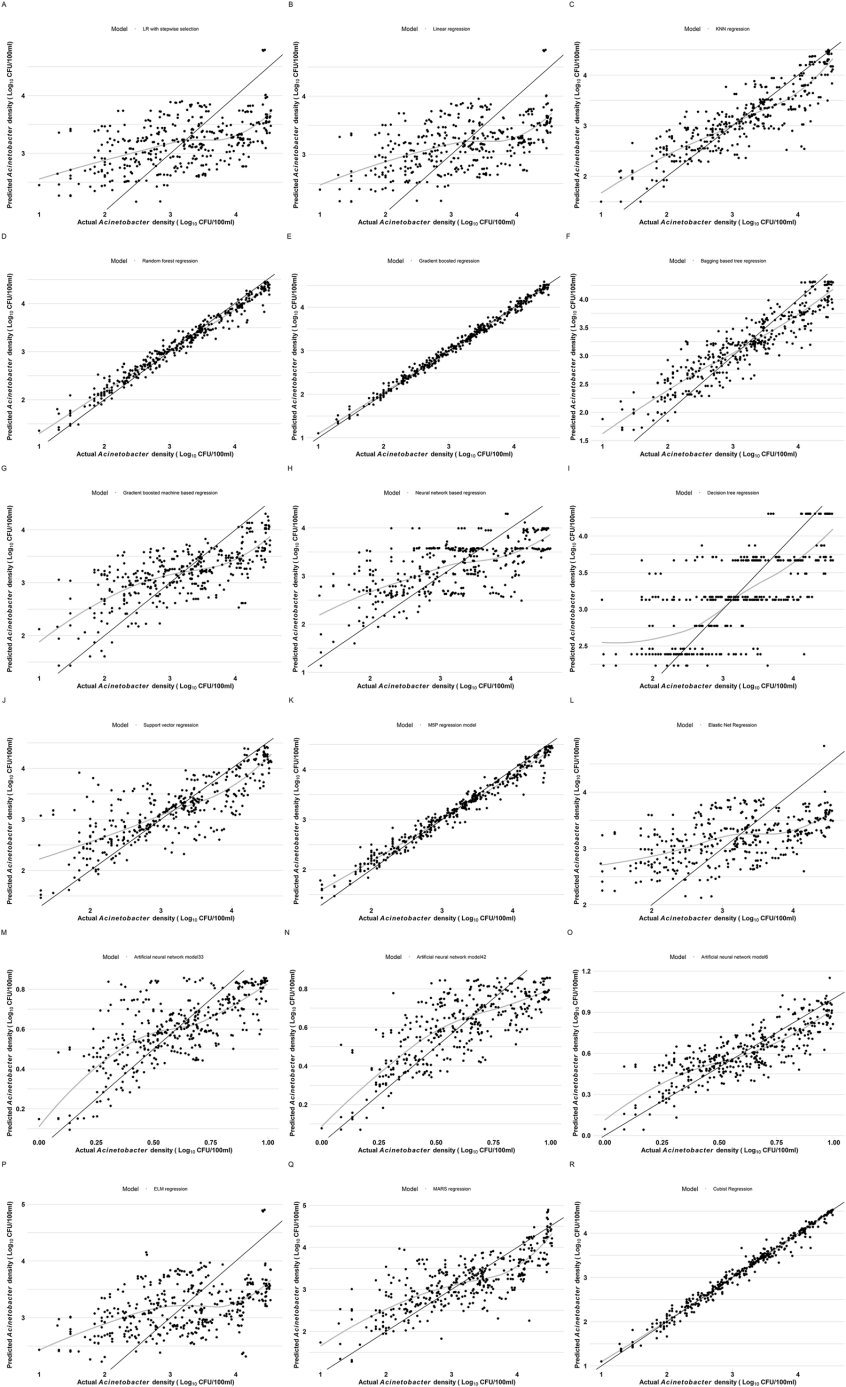
Comparison between actual and predicted AD by the eighteen ML models.

Figure 3 shows the residual diagnostics plots of the models comparing actual AD and forecasted AD values by the models. The observed results showed that actual AD and predicted AD value in the case of LR (A), LRSS (B), KNN (C), BRT 9F), GBM (G), NNT (H), DTR (I), SVR (J), ENR (L), ANET33 (M), ANER64 (N), ANET6 (O), ELM (P) and MARS (Q) skewed, and the smoothed trend did not overlap. However, actual AD and predicted AD values experienced more alignment and an approximately overlapped smoothed trend was seen in RF (D), XGB (E), M5P (K), and Cubist (R). Among the models, RF (D) and M5P (K) both overestimated and underestimated predicted AD at lower and higher values, respectively. Whereas XGB and Cubist both overestimated AD value at lower value with XGB closer to the smoothed trend that Cubist. Generally, a smoothed trend overlapping the gradient line is desirable as it shows that a model fits all values accurately/precisely.

The comparison of the partial-dependence profiles of PVs on AD prediction by the 18 modes using a unitary model by PVs presentation for clarity is shown in Figs. S2–S7. The partial-dependence profiles existed in i. a form where an average increase in AD prediction accompanied a PV increase (upwards trend), (ii) inverse trend, where an increase in a PV resulted in a decline AD prediction, (iii) horizontal trend, where increase/decrease in a PV yielded no effects on AD prediction, and (iv) a mixed trend, where the shape switch between 2 or more of i–iii. The models' response varied with a change in any of the PV, especially changes beyond the breakpoints that could decrease or increase AD prediction response.

The partial-dependence profile (PDP) of DO for models has a downtrend either from the start or after a breakpoint(s) of nature ii and iv, except for ELM which had an upward trend (i, Fig. S2). TEMP PDP had an upward trend (i and iv) and, in most cases filled with one or more breakpoints but had a horizontal trend in LRSS (Fig. S3). SAL had a PDP of a typical downward trend (ii and iv) across all the models (Fig. S4). While pH displayed a typical downtrend PDP in LR, LRSS, NNT, ENR, ANN6, a downtrend filled with different breakpoint(s) was seen in RF, M5P, and SVR; other models showed a typical upward trend (i and iv) filled with breakpoint(s) (Fig. S5). The PDP of TSS showed an upward trend that returned to a plateau (DTR, ANN33, M5P, GBM, RF, XFB, BRT), after a final breakpoint or a declining trend (ANNT6, SVR; Fig. S6). The BOD PDP generally had an upward trend filled with breakpoint(s) in most models (Fig. S7).

## Discussion

The present investigation studied the invaluableness of MLs in determining AD in waterbodies to shorten the turnaround time involved in routine determination of the emerging pathogen with significant public health priority and high case-fatality ratio. Jiang et al. previously demonstrated that ML models predicted and offered cost-effective risk assessment options for *Vibrio* spp. relative abundances on microplastics in the estuarine milieu based on easy-to-measure environmental variables^30^.

### Characteristics of the waterbodies

The pH of the waterbodies (5.05–9.11) did not satisfied South African water guidelines for irrigation purposes and recreational use of a pH range of 6.5–8.4 and 6.5–8.5, respectively^36^ but the average pH (7.76 ± 0.02) of the waterbodies met the FAO criteria^37^. In relation to the pathogen, *Acinetobacter* spp. are known to possess and survive under a wide pH (5–10) and temperature (− 20 to 44 °C) range with an optimal long-term survival temperature of 4–22 °C no matter nutrient availability^38^.

The observed EC (47.00–561.00 µS/cm) of the waterbodies generally satisfied the WHO guidelines for 2500 μS/cm in surface waters^39^, and the mean (218.66 ± 4.76 μS/cm) was in accepted limits of 400 µS/cm and 700 to 3000 µS/cm WHO and FAO standard for irrigation water^37^. The EC of the waterbodies also fell in the categories of Class I (excellent: ≤ 250 µS/cm) and Class II (good: 250–750 µS/cm) irrigation water EC limits classification^40^. The EC concentrations of the waterbodies will generally impact fishing negatively, as an EC range of 0.15–0.50 μS/cm are necessary to support fisheries according to the USEPA (United States Environmental Protection Agency)^41^.

TDS summed up organic and inorganic substances in the waterbodies but generally did not exceed the WHO’s maximum permissible limit of 1000 mg/L TDS in drinking water^39^. The TDS (23.00–279.00 mg/L) of the waterbodies followed the World Health Organization standard of a TDS < 300 mg/L (excellent) and its average (110.53 ± 2.36 mg/L) does not exceed the USEPA and WHO limit for drinking water (500 mg/L)^41,42^.

However, the TBS average values of the waterbodies exceeded the WHO guideline of 5 NTU^39^. Higher EC, TDS, and TBS in surface waters are generally attributed to wastewater and anthropogenic activities inputs^43^. Also, high levels of EC, TDS and TBS are known to impair visibility, cleanliness, safety, aesthetics, and recreational use of river waters^44^. The mean TSS (80.17 ± 5.09 mg/L) of the waterbodies exceeded the WHO (2006) wastewater discharge limit of 60 mg/L and exceeded the Australia and New Zealand (2000) guideline limits (TSS < 0.03 mg/L) of water quality for aquaculture^45,46^. In addition, the average BOD level (4.00 ± 0.10 mg/L) of the waterbodies complied with the tolerance limit of 5 mg/L in surface waters for aquatic life^47^. Higher level of BOD in waterbodies depletes DO available for aquatic organisms^48^ and generally have negative impacts on fishing and fish harvest.

The average AD (3.19 ± 0.03 log CFU/100 mL) obtained in this study is comparable to AD reported from waterbody impacted by hospital wastewater, WWTP, informal settlements, and veterinary clinics effluents along Umhlangane River course in Durban South Africa^49^. The observed DO (8.82 ± 0.04 mg/L) and BOD (4.00 ± 0.10 mg/L) both suggested the facultative aerophilic characteristics of *Acinetobacter* and a relatively high nutrient composition of the rivers’ probable from wastewater effluents. The average EC in the waterbodies was 218.66 ± 4.76 µS/cm. This shows high level of organic carbon (DOC) in the rivers. EC is an indirect indicator of DOC^25,50,51^ and found to have associations with *Acinetobacter*-specific ARG and other ARG abundance^25,52,53^. Generally, *A. baumannii* in the environment can survive irrespective of the level of DO^54^.

The finding from this study revealed that AD negligible—positive but very weak—correlated with pH (r = 0.03), and SAL (r = 0.06) and—negatively—with TDS (r = − 0.05) and EC (r = − 0.04) (Table 2). These results can be attributed to the ability of the *Acinetobacter* to survive under a wide range of harsh environmental conditions. A significantly positive correlation between AD and BOD (r = 0.26), TSS (r = 0.26), and TBS (r = 0.26) indicated a considerable increase AD with an increase in nutrient and DOC pollution in aquatic environments (Fig. S7). Also, findings showed a moderate positive correlation between TEMP and AD (r = 0.43), suggesting that AD improves in abundance with an increase in temperature^38^ to specific breakpoints. AD moderately and inversely correlated with DO (r = − 0.46), indicating that *Acinetobacter* abundance increases with an anaerobic condition or low oxygen level.

### Model predicted AD and explanatory contribution of PVs

The predicted AD average and range values by the 18 ML models differed. The present study's findings suggested that both lower/upper bound and the general trend characteristic of the prediction is far more important than the average prediction only. Most algorithms had higher average predictions but overestimated or underestimated AD values at lower and upper bounds, respectively. Thus, algorithms other than XGB and Cubist are not suitable for predicting AD in waterbodies. Whereas the performance of most ML algorithms, such as RF, DTR, and MARS^43,55^, has been praised in terms of average predictions and regression metrics, most studies neglect consideration of the lower/upper bound and the general trend characteristic of their predictions—which are far significant when dealing with infectious organisms/poison that might have low infectivity dose/potent at a very low concentration. Several researchers also reported the superiority of XGB against several ML algorithms in predictive performance in terms of average prediction, and sensitivity^43,55^. Although a previous study showed that RF models achieved higher level of accuracy than XGB, SVR, and ENR in predicting the *Vibrio* spp. relative abundance on microplastics, the actual trend characteristics including the lower/upper bounds were not reported^30^. The difference in the models’ trend coverage and boundary characteristics in AD predictions are attributable to the capability of the models to capture the complex interactions of co-occurrence levels/changes in different environmental variables at different degrees or concentrations. The performance of Cubist [3.1736 (1.1012–4.5300)] was also found to be comparable to XGB [3.1792 (1.1040–4.5828)] in term of trend and boundaries characteristics as both models outshined other models. A typical problem with most algorithms observed in this study was over-estimation and underestimation of AD at lower and higher concentrations, respectively. These limitations suggested that the models could raise false alarm of high risk at lower AD as well as undermine higher risk at higher concentrations of AD. An indication that those models could not capture the nonlinear complex relationships between AD, PVs, and underlying anthropogenic inputs.

Nevertheless, the absolute contributions of individual PV change to models’ prediction of AD from their models attributed mean values varied (Fig. 2). The behaviours could be interpreted in term of the complex interactions among the PVs coupled with the prevailing anthropogenic fluxes in the waterbodies. Several PVs undergo fluctuations co-concurrently unlike behaviours in models in which other PVs are held constant to assess a particular PV’s effects on the outcome variable (AD). These interactions are capture to some great degrees by the algorithms leading to differences in the ranking of PVs contributions to AD predictions by the algorithms. Also, intrinsic characteristics of the distinct algorithms and data noise are major causes of differences in observed contributions of variables in ML models^30^.

Considering the overall performance of 18 AI-based models assayed in this study using four metrics, XGB (MSE = 0.0059, RMSE = 0.0770; R^2^ = 0.9912; MAD = 0.0440) and Cubist (MSE = 0.0117, RMSE = 0.1081, R^2^ = 0.9827; MAD = 0.0437) were the best models ranking in first and second position respectively, to outshined others in AD prediction in waterbodies (Table 4). XGB has reputation of been the best performer ML algorithms in most microbiological regression studies compared with others^30^. Cubist has been demonstrated to outperformed partial least squares, RF, and MARS in predicting soil property including soil total nitrogen, organic carbon, total sulphur, exchangeable calcium clay; sand, and cation exchange capacity, and pH and RF, classification, and regression trees, SVM, and KNN predicting NH_4_–N and COD in subsurface constructed wetlands effluents^56,57^. In forecasting daily dissemination of COVID-19 vaccination, Cubist outperformed ENR, Gaussian Process, Slab (SPIKES), and Spikes ML algorithms^58^. Also, Cubist has been shown to outmatched XGB in predicting left ventricular pressures, volumes, and stresses^59^. An ensemble of XGB and Cubist could be further exploited for a better performance in forecasting AD in waterbodies. However, ANN (R^2^ = 0.953) was demonstrated to show a superior predictive coefficient over Cubist model (R^2^ = 0.946) and LR (R^2^ = 0.481) when assaying faecal coliform content in treated wastewater for reuse purposes^60^. Generally, while XGB involved ensemble of trees that capture multidimensional interactions/relationships, Cubist combined the strengths of both linear regression equations and a committee tree-based structural nodes for capture effectively linear and nonlinear multidimensional relationships among variables and outcome event^56^. The results show that ANET6, ANRT42, ANET33, M5P, and RF had MSE and RMSE that placed them in the 3, 4, 5, 6, and 7 position among the MLs in predicting AD, their performances are to be avoided for practical forecast of AD for preventive purposes.

**Table 4:** Predictive performance of eighteen regression algorithms in predicting AD in the waterbodies.

Rank	ML	MSE	ML	RMSE	ML	R^2^	ML	MAD
1	XGB	0.0059	XGB	0.0770	XGB	0.9912	Cubist	0.0437
2	Cubist	0.0117	Cubist	0.1081	Cubist	0.9827	XGB	0.0440
3	ANET6	0.0172	ANET6	0.1310	M5P	0.9589	ANET6	0.0856
4	ANRT42	0.0220	ANET42	0.1483	RF	0.9584	M5P	0.0863
5	ANET33	0.0253	ANET33	0.1590	BRT	0.8140	ANET33	0.0987
6	M5P	0.0275	M5P	0.1657	KNN	0.7459	RF	0.1044
7	RF	0.0282	RF	0.1679	ANET6	0.6727	ANET42	0.1078
8	BRT	0.1261	BRT	0.3551	SVR	0.6294	SVR	0.2142
9	KNN	0.1723	KNN	0.4150	MARS	0.5913	KNN	0.2297
10	SVR	0.2475	SVR	0.4975	ANET42	0.5804	BRT	0.2385
11	MARS	0.2770	MARS	0.5263	DTR	0.5460	DTR	0.3146
12	DTR	0.3032	DTR	0.5506	ANET33	0.5178	MARS	0.3176
13	GBM	0.3547	GBM	0.5955	GBM	0.4768	GBM	0.4148
14	NNT	0.3834	NNT	0.6192	NNT	0.4259	NNT	0.4399
15	ENR	0.4853	ENR	0.6967	ENR	0.2732	LRSS	0.5421
16	LR	0.5036	LR	0.7097	LR	0.2570	LR	0.5774
17	LRSS	0.50506	LRSS	0.7107	LRSS	0.2549	ENR	0.6104
18	ELM	0.5447	ELM	0.7380	ELM	0.1965	ELM	0.6368

### Feature importance of PVs in predicting AD

TEMP was the most important PV in predicting AD in the waterbodies and ranked by 10/18 ML-algorithms including RF, XGB, Cubist, BRT, and NNT accounting 45.90%, 43.00%, 50.82%, 44.87%, and 68.77% in respective models, as well as 82.31%, 83.30%, 57.00%, 50.58%, and 57.58% RMSE dropout loss in ANET42, ANET10, ELM, M5P, and DTR respectively. The observed results can be explained in term of the direct and indirect influence TEMP had on other PVs and AD in the waterbodies. DO decreases with increase temperature, favoured facultative aerobic lifestyle of *Acinetobacter*. Also, temperature increase decomposition of organic matters in waterbodies, thereby leading to high BOD contents providing more nutrients for AD and other microbial lives. Resultant increase in DOC in waterbodies is an indirect indicator of EC^25,50,51^ and found to have associations with *Acinetobacter*-specific ARG abundance in waterbodies^25,52,53^. BOD was another significant feature identified in forecasting AD in the waterbodies and ranked first in 3/18 [MARS (182.96%), LR (27.42%), and GBM (22.35%)] and second in 8/18 models [KNN (42.69%), XGB (38.60%); BRT (32.51%), ELM (23.17%), SVR (35.77%), DTR (35.13%), ENR (21.84%) and LRSS (31.65%)]. BOD is a measure of nutrient pollution from anthropogenic inputs such as wastewater effluents, agricultural activities, and environmental events such as rainwater runoffs among others. BOD also influence EC, TDS, and TBS in surface waters^43^ Whereas SAL was identified as first important feature in in 2/18 (KNN, ANET33) and second in 3/18 (Cubist, ANET42, ANET6) models, *Acinetobacter* can only survive relatively high SAL without improving its population density (Fig. S4). Unlike *Vibrio* spp, whose high density are linked with high salinity^30^ as it promotes genes expression and functional proteins^61^ and eventual vibrio growth and reproduction^62^, high SAL are not suitable for AD as its inhibitory for growth related gene expression.

The sensitivity analyses of the 18 ML predictive models of AD using the residual diagnostics plots found that LR (A), LRSS (B), KNN (C), BRT (F), GBM (G), NNT (H), DTR (I), SVR (J), ENR (L), ANET33 (M), ANER64 (N), ANET6 (O), ELM (P) and MARS (Q) did not fit the data optimally. This imply that the models are not suitable for forecasting AD in waterbodies. Meanwhile models such as RF (D), XGB (E), M5P (K), and Cubist (R) fitted the data with more alignment and approximately overlapped smoothed trend between the actual and the predicted AD values, RF (D) and M5P (K) over-predicted and under-predicted AD at lower and higher extremities, respectively. Thus, could be interpreted as forecasting exaggerated risk (AD) at probable innocuous level while weakening true risk at higher extremity. Such models are not suitable to assess real life events of AD in waterbodies. Although both XGB and Cubist predicted AD value slightly higher than the actual value at lower extremities, XGB had a closer fit smoothed trend than Cubist. Compared to other models assayed in this study, the duo is the best and could be applied for AD AI-smart system design for water quality monitoring. A stacked model of XGB and Cubist may outmatch and overcome the limitation the two models had at the lower extremity of AD value.

The overall summary of the PDPs of the PVs on AD prediction by the 18 modes (Figs. S2–S7), found that any degree of change/flux in a particular PV especially changes beyond its breakpoints attracted a corresponding varied response in AD which could decrease or increase AD prediction response. The various forms of partial-dependence profiles as explained in previous section also showed the direct/indirect/complex interactions between a PV and AD coupled with the sensitivity of a model in mapping the relationships. Summarily, the increase in AD level (PDP) in most models equivalent to a decline trend in DO and SAL especially after its breakpoint(s) excluding ELM where DO had upward trend (i; Figs. S2 and S4). These patterns revealed a nonlinear relationship between AD and the PVs. A near increase-by-increase relationship exist between TEMP and AD in most models coupled with one or more breakpoints. LRSS revealed a zero-relationship between AD and TEMP indicating its inability to map the relationship between them. Although *Acinetobacter* has been showed to have a broad pH range, a typical downtrend PDP of pH by LR, LRSS, NNT, ENR, ANN6—filled with breakpoint(s) in RF, M5P, and SVR while other models showed a typical upward—is informative of the weakness of the models as increasing in pH from 5.02 to 10 promotes *Acinetobacter* growth^38^. AD prediction responses aligned with a general increase in BOD regardless of breakpoint(s) in most models revealed important of nutrients for *Acinetobacter* population density in waterbodies.

Furthermore, the strengths of this current study aside been the first that assessed AD in waterbodies receiving hospital and municipal wastewater effluents along their courses, two ML algorithms optimally and accurately predict AD, proven to be promising candidates for developing SAIS for AD determination and thereby shorten the turnaround time and reduce labour involved in experimental approaches. Also, the MLs were able to capture nonlinear complex multidimensional interactions between AD and PVs as well as their inherent anthropogenic fuels which conventional mathematical models could not robustly mapped^63^. In addition, the MLs are amenable to improvements and can be utilized across several water management landscape. However, the shortcoming of the present study lies in the lack of spatiotemporal covariates that could improve upon the ML models’ predictions as stochastic distributions of waterborne pathogens are governed by both spatial extension and temporal duration across depth in water columns. Future studies should seek data from a wide range of socioeconomic activities/areas as well as include spatiotemporal and geospatial inputs in developing AI-based predictive framework for AD determination.

## Conclusion

The present study has proven SAIS as an evidence-based strategy to shorten the turnaround time involved in assessing AD in waterbodies; thereby minimizing exposure. The best models (XGB/Cubist) identified in this study could be developed into standalone SAIS (XGB/Cubist, XGB-Cubist ensemble, or web app) or integrated into existing instrumentations for PV estimation in waterbodies to enhance timely decision-making of microbiological qualities of waterbodies for irrigation and other purposes. The study also unveiled temperature and BOD as significant candidates for predicting AD in waterbodies in most models. Finally*,* AD in waterbodies could accurately and reliably predicted via AI-based smart systems that rely on waterbody physicochemical variables’ dynamics in a low-cost and time-effective manner.

## Supplementary Information

Supplementary Information.

## Data Availability

All data generated or analysed during this study are included in this published article and its Supplementary Information Files.

## Abbreviations

AD: *Acinetobacter* density
ANN: Artificial neural network
BOD: Biochemical oxygen demand
BRT: Boosted regression tree
Cubist: Cubist regression
DTR: Decision tree regression
DO: Dissolved oxygen
ENR: Elastic net regression
EC: Electrical conductivity
XDR: Extensively drug-resistant
XGB: Extreme gradient boosted regression
ELM: Extreme learning machine
GBM: Gradient boosted machine
ISW: Irrigation source waters
KNN: K-nearest neighbours
LR: Linear regression
LRSS: Linear regression with stepwise selection
ML: Machine learning
MSE: Mean squared error
MAD: Median absolute deviation
MDR: Multidrug-resistant
MARS: Multivariate adaptive regression splines
MHWE: Municipal and hospital wastewater effluents
NNT: Neural network
PVs: Physicochemical variables
RF: Random forest
RMSE: Root-mean-squared error
SAL: Salinity
SAIS: Smart artificial intelligent system
SVR: Support vector regression
TEMP: Temperature
TDS: Total dissolved solids
TBS: Turbidity
WWTPs: Wastewater treatment plants
