# Supplementary material for: Machine learning-guided determination of Acinetobacter density in waterbodies receiving municipal and hospital wastewater effluents
Source: Sci Rep. 2023 May 12;13:7749. doi: 10.1038/s41598-023-34963-6 (PMC10177717; doi:10.1038/s41598-023-34963-6)
Supplement: Supplementary file 1 — Supplementary Information. [file 41598_2023_34963_MOESM1_ESM.docx]

**Supplementary methods and results for** “**Machine learning-guided determination of *Acinetobacter* density in waterbodies receiving municipal and hospital wastewater effluents**”

**Methods**

**ML model fine tuning and hyperparemeters**

1. LRSS

.outcome ~ pH + SAL + TEMP + TSS + DO + BOD

Start: AIC=-227.23

Step: AIC=-247.57

1. KNN


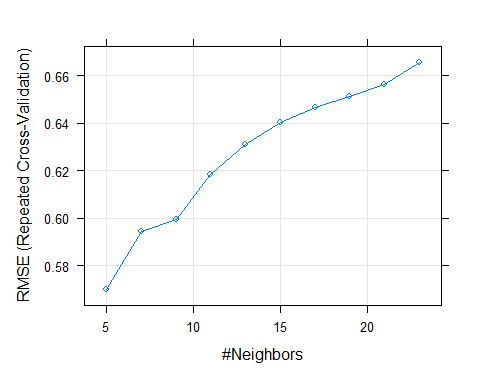


RF


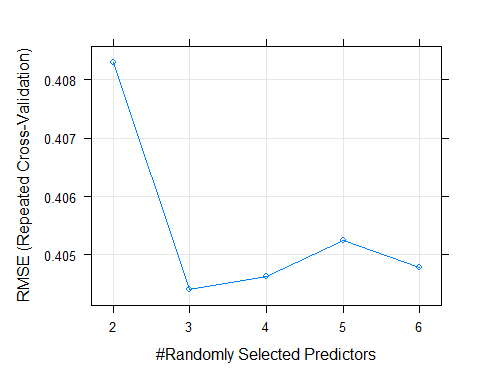


Gradient boosted regression

RMSE was used to select the optimal model using the smallest value.

The final values used for the model were nrounds = 150, max_depth = 3,

eta = 0.3, gamma = 0, colsample_bytree = 0.8, min_child_weight = 1

and subsample = 0.75.

## Tuning parameter 'gamma' was held constant at a value of 0

## Tuning

## parameter 'min_child_weight' was held constant at a value of 1

## RMSE was used to select the optimal model using the smallest value.

## The final values used for the model were nrounds = 150, max_depth = 3, eta

## = 0.3, gamma = 0, colsample_bytree = 0.8, min_child_weight = 1 and subsample

## = 0.75.

# # Best tuning parameter mtry

model_xgb$bestTune

## nrounds max_depth eta gamma colsample_bytree min_child_weight subsample

## 51 150 3 0.3 0 0.8 1 0.75

mars

RMSE was used to select the optimal model using the smallest value.

The final values used for the model were nprune = 20 and degree = 4.


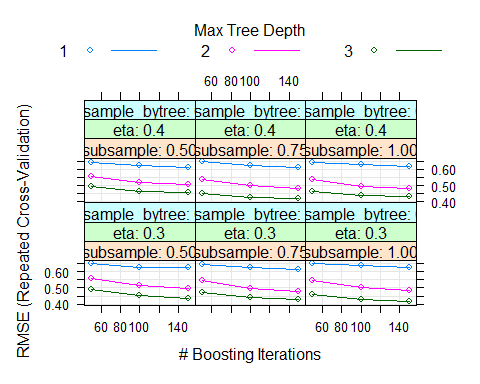

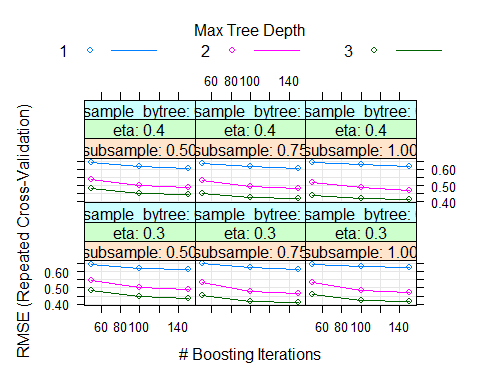


**BRT**

summary(model_brt

y = 380, X = 6, mtrees 25, OOB 1, comb 1, call 5

GBM

summary(model_gbm)


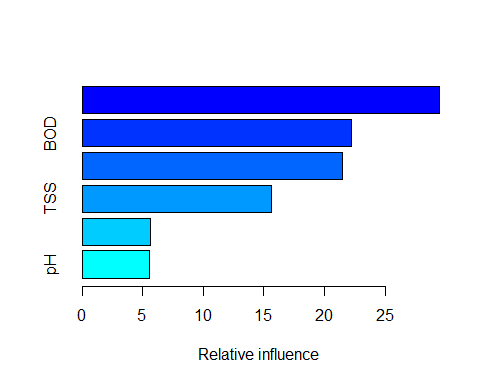


## var rel.inf
## TEMP TEMP 29.512370
## BOD BOD 22.263625
## DO DO 21.464021
## TSS TSS 15.600376
## SAL SAL 5.625861
## pH pH 5.533747


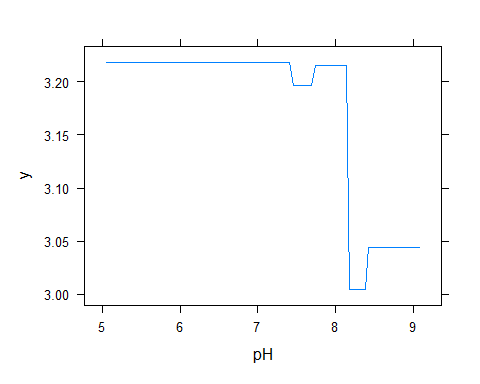


NNET

## a 6-6-1 network with 49 weights
## options were - linear output units decay=0.1
## b->h1 i1->h1 i2->h1 i3->h1 i4->h1 i5->h1 i6->h1
## -0.06 -1.08 -0.76 -0.51 0.71 0.77 0.77
## b->h2 i1->h2 i2->h2 i3->h2 i4->h2 i5->h2 i6->h2
## -0.20 -0.90 -0.31 -0.28 -0.42 1.90 -1.60
## b->h3 i1->h3 i2->h3 i3->h3 i4->h3 i5->h3 i6->h3
## 0.27 -3.58 -1.25 1.25 0.01 0.52 0.32
## b->h4 i1->h4 i2->h4 i3->h4 i4->h4 i5->h4 i6->h4
## 0.00 -0.18 0.11 0.79 -0.01 -0.36 0.70
## b->h5 i1->h5 i2->h5 i3->h5 i4->h5 i5->h5 i6->h5
## -0.53 -1.47 -0.08 1.38 -0.65 -0.62 -0.87
## b->h6 i1->h6 i2->h6 i3->h6 i4->h6 i5->h6 i6->h6
## -0.15 -1.91 0.28 1.94 -0.22 -0.12 2.24
## b->o h1->o h2->o h3->o h4->o h5->o h6->o
## 2.22 -1.60 -1.00 0.95 2.43 -1.85 -0.42

DTR


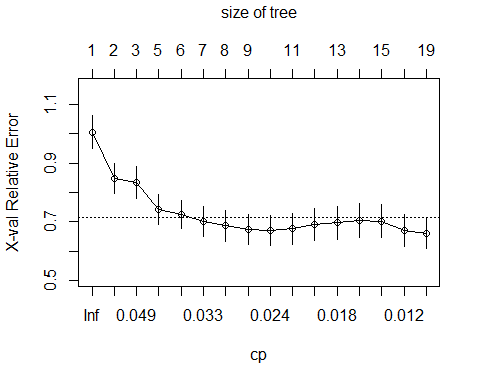


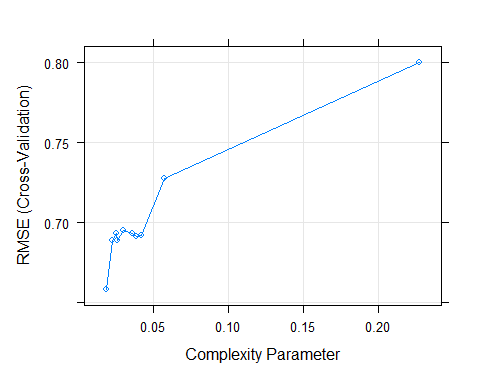


SVR

SVM-Type: eps-regression, SVM-Kernel: radial; cost: 1; gamma: 0.1666667; epsilon: 0.1

M5P


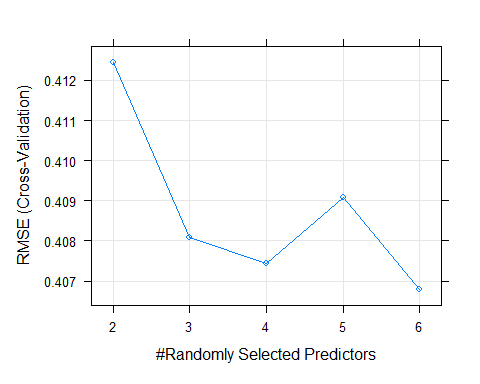


ENR


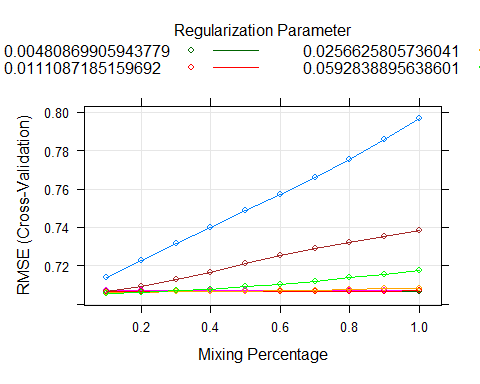


ANET33

$result.matrix

[,1]

error 4.777379e+00

reached.threshold 9.976136e-03

steps 9.904000e+03

Intercept.to.1layhid1 -5.437419e-01

pH.to.1layhid1 -1.565035e+00

SAL.to.1layhid1 -4.854390e-01

TEMP.to.1layhid1 -3.898728e+00

TSS.to.1layhid1 3.332851e+00

DO.to.1layhid1 1.912399e+00

BOD.to.1layhid1 2.593676e+00

Intercept.to.1layhid2 6.489725e+00

pH.to.1layhid2 3.415862e+00

SAL.to.1layhid2 8.418756e+00

TEMP.to.1layhid2 7.188007e+00

TSS.to.1layhid2 -1.520425e+01

DO.to.1layhid2 -1.478994e+01

BOD.to.1layhid2 -1.681048e+00

Intercept.to.1layhid3 1.664179e+00

pH.to.1layhid3 -1.526164e+00

SAL.to.1layhid3 6.984581e+00

TEMP.to.1layhid3 -6.954129e+00

TSS.to.1layhid3 -2.312118e+01

DO.to.1layhid3 3.184074e+00

BOD.to.1layhid3 -5.464162e-02

Intercept.to.2layhid1 -3.305125e+00

1layhid1.to.2layhid1 4.488590e+01

1layhid2.to.2layhid1 1.311472e+00

1layhid3.to.2layhid1 -1.938816e+01

Intercept.to.2layhid2 -4.737584e+00

1layhid1.to.2layhid2 7.833783e+01

1layhid2.to.2layhid2 9.264443e-02

1layhid3.to.2layhid2 -3.482285e+01

Intercept.to.2layhid3 -1.492480e+00

1layhid1.to.2layhid3 4.552868e+00

1layhid2.to.2layhid3 3.530304e+00

1layhid3.to.2layhid3 -3.647379e+00

Intercept.to.Acineto 1.196209e-01

2layhid1.to.Acineto -1.011271e+00

2layhid2.to.Acineto 9.510550e-01

2layhid3.to.Acineto 8.213007e-01

ANET42

$result.matrix

[,1]

error 4.156842e+00

reached.threshold 8.811814e-03

steps 5.883000e+03

Intercept.to.1layhid1 -1.049487e+01

pH.to.1layhid1 9.550651e+00

SAL.to.1layhid1 -5.123225e+01

TEMP.to.1layhid1 1.153924e+00

TSS.to.1layhid1 4.632863e+01

DO.to.1layhid1 1.084746e+00

BOD.to.1layhid1 9.542402e+00

Intercept.to.1layhid2 -1.407508e+00

pH.to.1layhid2 2.043731e+00

SAL.to.1layhid2 -1.848276e+00

TEMP.to.1layhid2 -1.806560e+00

TSS.to.1layhid2 2.572207e-03

DO.to.1layhid2 -6.548291e-01

BOD.to.1layhid2 4.002404e+00

Intercept.to.1layhid3 2.101725e+00

pH.to.1layhid3 -1.171262e+01

SAL.to.1layhid3 1.100721e+01

TEMP.to.1layhid3 1.397552e+01

TSS.to.1layhid3 1.249279e+01

DO.to.1layhid3 2.517133e+00

BOD.to.1layhid3 -1.463478e+01

Intercept.to.1layhid4 1.219433e+01

pH.to.1layhid4 8.885284e+00

SAL.to.1layhid4 1.302115e+01

TEMP.to.1layhid4 3.973669e+01

TSS.to.1layhid4 -1.527712e+02

DO.to.1layhid4 -5.238261e+01

BOD.to.1layhid4 2.104306e+01

Intercept.to.2layhid1 1.034696e-01

1layhid1.to.2layhid1 -2.942211e+00

1layhid2.to.2layhid1 -2.472882e+00

1layhid3.to.2layhid1 -1.041343e+00

1layhid4.to.2layhid1 -2.708068e+00

Intercept.to.2layhid2 7.399394e-01

1layhid1.to.2layhid2 -1.866493e+02

1layhid2.to.2layhid2 -7.829317e+00

1layhid3.to.2layhid2 -4.484255e+00

1layhid4.to.2layhid2 3.518068e+00

Intercept.to.Acineto 8.581403e-01

2layhid1.to.Acineto -2.357882e+00

2layhid2.to.Acineto -1.945739e+00

ANET6

$result.matrix

[,1]

error 3.242534e+00

reached.threshold 9.793532e-03

steps 3.538800e+04

Intercept.to.1layhid1 4.343533e+00

pH.to.1layhid1 -2.633151e+00

SAL.to.1layhid1 -6.683505e+00

TEMP.to.1layhid1 -9.446165e+00

TSS.to.1layhid1 -1.665197e+01

DO.to.1layhid1 1.029247e+01

BOD.to.1layhid1 4.736946e+00

Intercept.to.1layhid2 -5.129649e+00

pH.to.1layhid2 6.064627e+00

SAL.to.1layhid2 -2.440785e+01

TEMP.to.1layhid2 1.835408e+00

TSS.to.1layhid2 -6.471240e+00

DO.to.1layhid2 1.882425e+00

BOD.to.1layhid2 2.649308e+00

Intercept.to.1layhid3 -3.778119e+00

pH.to.1layhid3 -4.835310e+00

SAL.to.1layhid3 3.300276e+01

TEMP.to.1layhid3 -3.161186e+01

TSS.to.1layhid3 2.613370e+01

DO.to.1layhid3 6.713434e+01

BOD.to.1layhid3 5.797129e+00

Intercept.to.1layhid4 -5.724932e+00

pH.to.1layhid4 4.655949e-01

SAL.to.1layhid4 -1.282121e+01

TEMP.to.1layhid4 -1.055600e+01

TSS.to.1layhid4 -7.908118e+00

DO.to.1layhid4 1.463542e+01

BOD.to.1layhid4 3.673359e+00

Intercept.to.1layhid5 3.281499e+00

pH.to.1layhid5 -3.538442e+00

SAL.to.1layhid5 -1.825608e+00

TEMP.to.1layhid5 -2.622971e+00

TSS.to.1layhid5 -4.245828e+00

DO.to.1layhid5 4.259153e+00

BOD.to.1layhid5 1.779192e+00

Intercept.to.1layhid6 -7.716578e+00

pH.to.1layhid6 7.918228e+00

SAL.to.1layhid6 -2.481435e-01

TEMP.to.1layhid6 5.623819e+00

TSS.to.1layhid6 5.134436e+00

DO.to.1layhid6 -1.080870e+01

BOD.to.1layhid6 -8.408232e-01

Intercept.to.Acineto -1.343955e+00

1layhid1.to.Acineto -1.245907e+00

1layhid2.to.Acineto 9.082713e-01

1layhid3.to.Acineto 6.734978e-01

1layhid4.to.Acineto -6.837441e-01

1layhid5.to.Acineto 2.629765e+00

1layhid6.to.Acineto 1.653165e+00


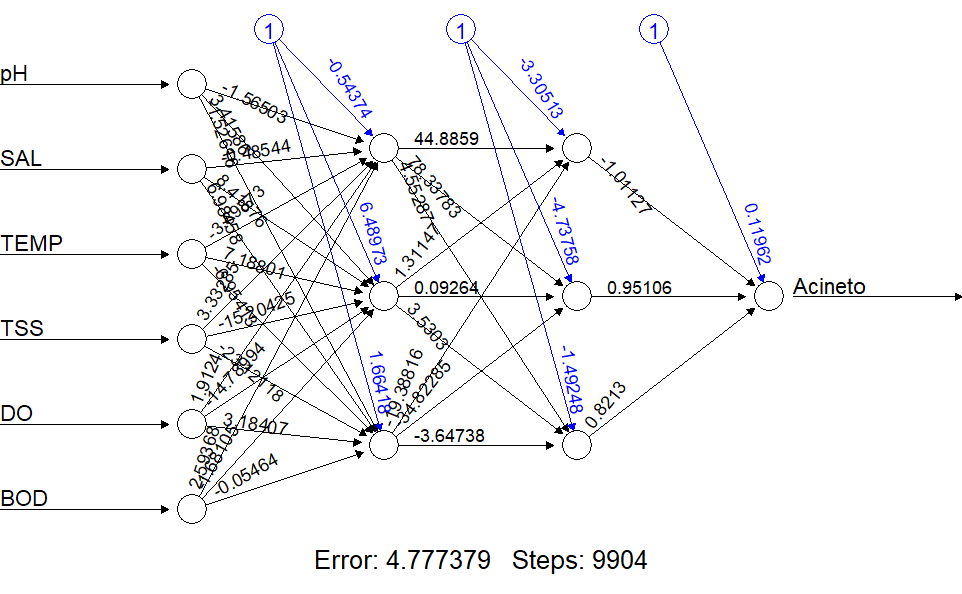


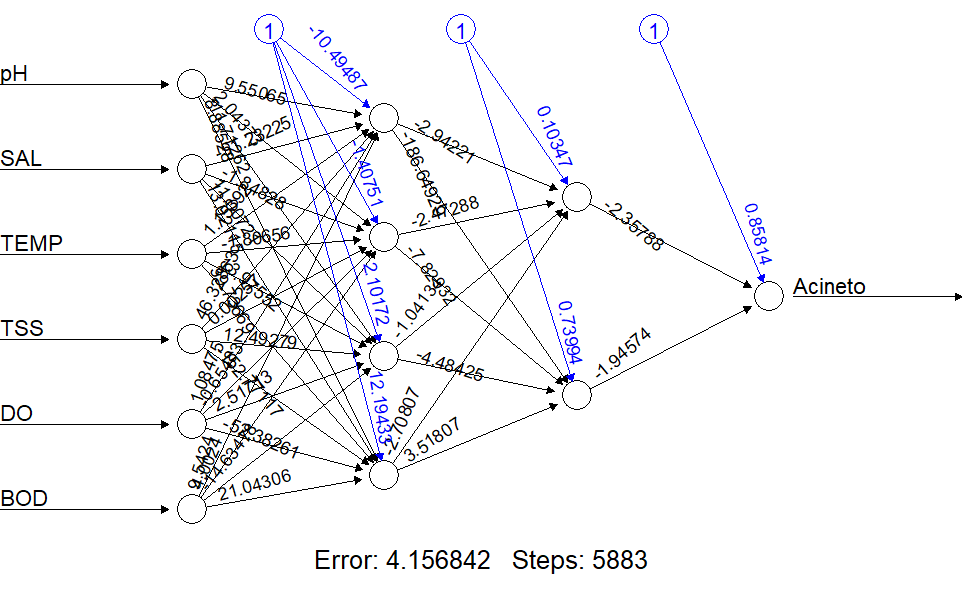


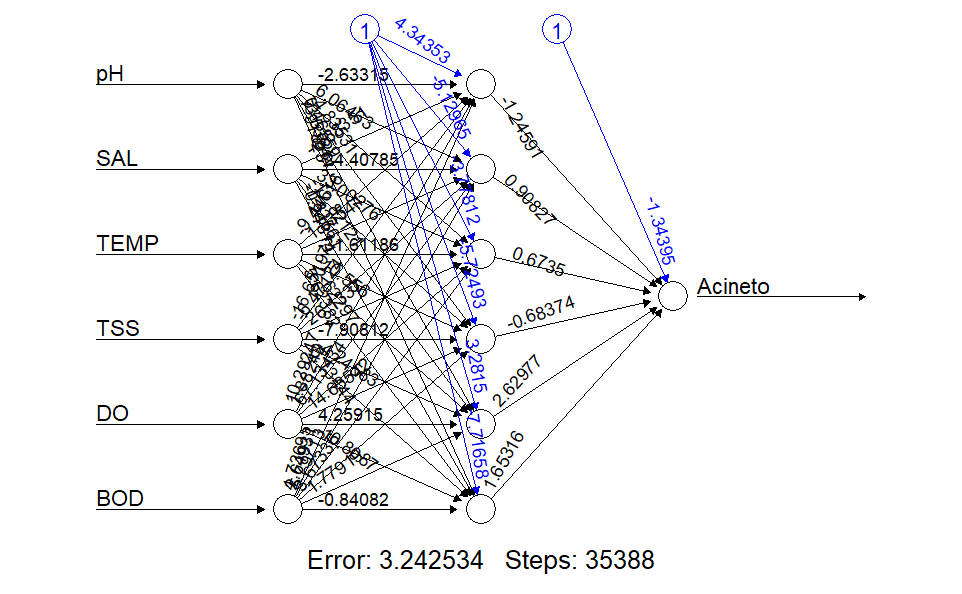


ELM

Extreme learning model, elm (regression):

call: elm(Acineto ~ ., data = train.data, nhid = 20, actfun = "purelin", init_weights = "normal_gaussian")
 hidden units : 20
 activation function: purelin
 mse : 0.5446799

MARS


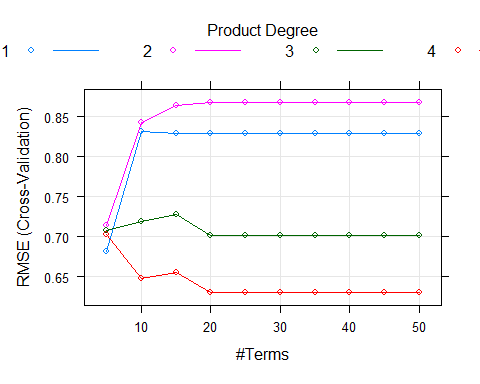


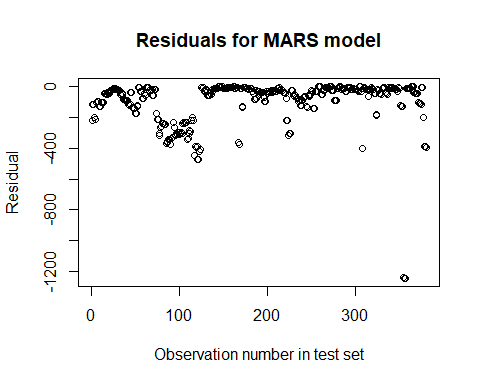


Selected 14 of 21 terms, and 5 of 6 predictors (nprune=20)

Termination condition: Reached nk 21

Importance: TEMP, BOD, SAL, TSS, DO, pH-unused

Number of terms at each degree of interaction: 1 2 6 4 1

GCV 0.3331699 RSS 105.2661 GRSq 0.511085 RSq 0.5913406

Cubist


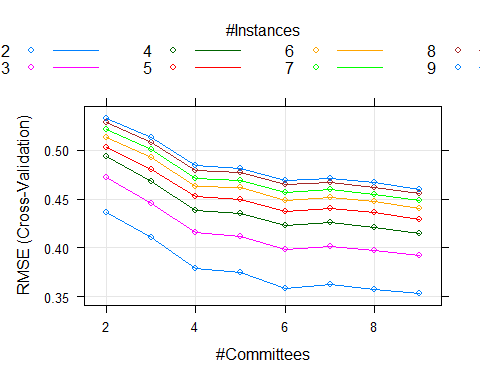


RMSE was used to select the optimal model using the smallest value.

The final values used for the model were committees = 9 and neighbors = 2.

Cubist

committees neighbors RMSE Rsquared MAE

9 2 0.3537615 0.8124143 0.2393605

Results

DTR


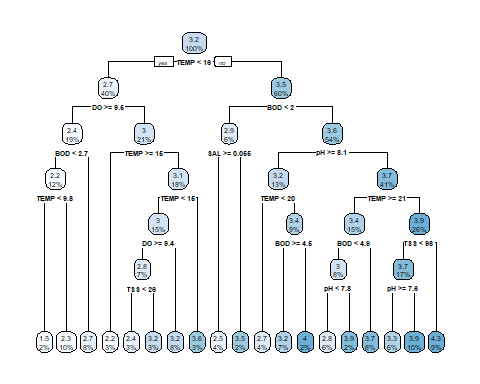


MARS results

coefficients
 (Intercept) 3.7082357
 h(0.453457-TEMP) = -1.3206949
 h(-0.661465-BOD) = -1.7483488
 h(-0.268654-SAL) * h(TEMP-0.453457) = -1.4050364
 h(SAL- -0.268654) * h(TEMP-0.453457) = -1.1211693
 h(-1.01793-SAL) * h(-0.661465-BOD) =7.0437330
 h(SAL- -1.01793) * h(-0.661465-BOD) =0.7537466
 h(0.453457-TEMP) * h(BOD-1.2978)= 1.1479762
 h(0.453457-TEMP) * h(1.2978-BOD) =0.3525903
 h(SAL- -1.01793) * h(TEMP- -0.340244) * h(-0.661465-BOD) =-1.1843070
 h(0.293301-SAL) * h(TSS- -0.254953) * h(BOD- -0.661465)=1.4816591
 h(SAL-0.293301) * h(TSS- -0.254953) * h(BOD- -0.661465)=1.2862669
 h(-0.859043-TEMP) * h(-0.254953-TSS) * h(BOD- -0.661465)=7.4532739
 h(TEMP- -0.859043) * h(-0.254953-TSS) * h(-0.452583-DO) * h(BOD- -0.661465)=3.9032858


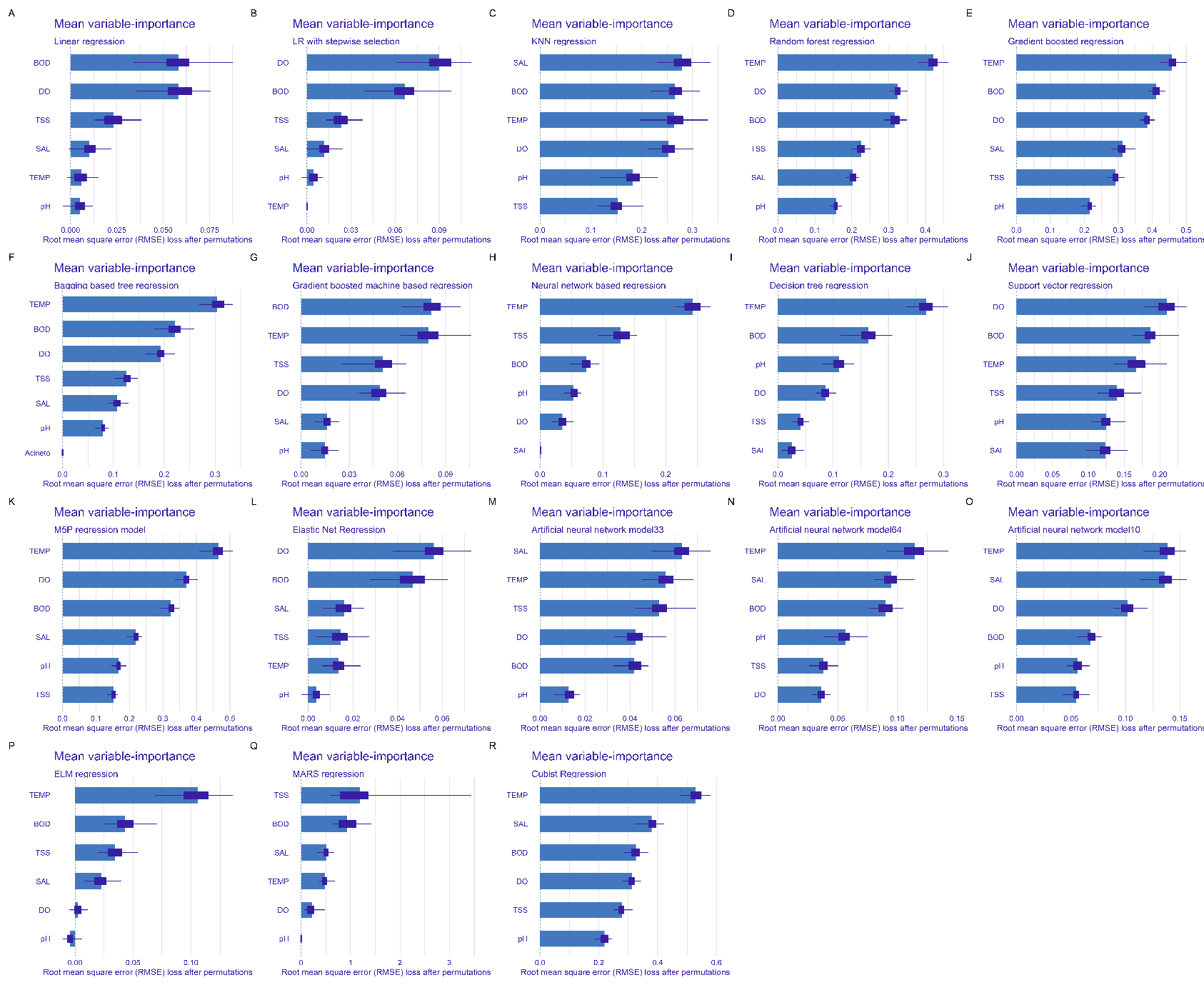


Figure S1. Average feature importance over 100 resampling permutational draws of PVs in predicting AD in the waterbodies.


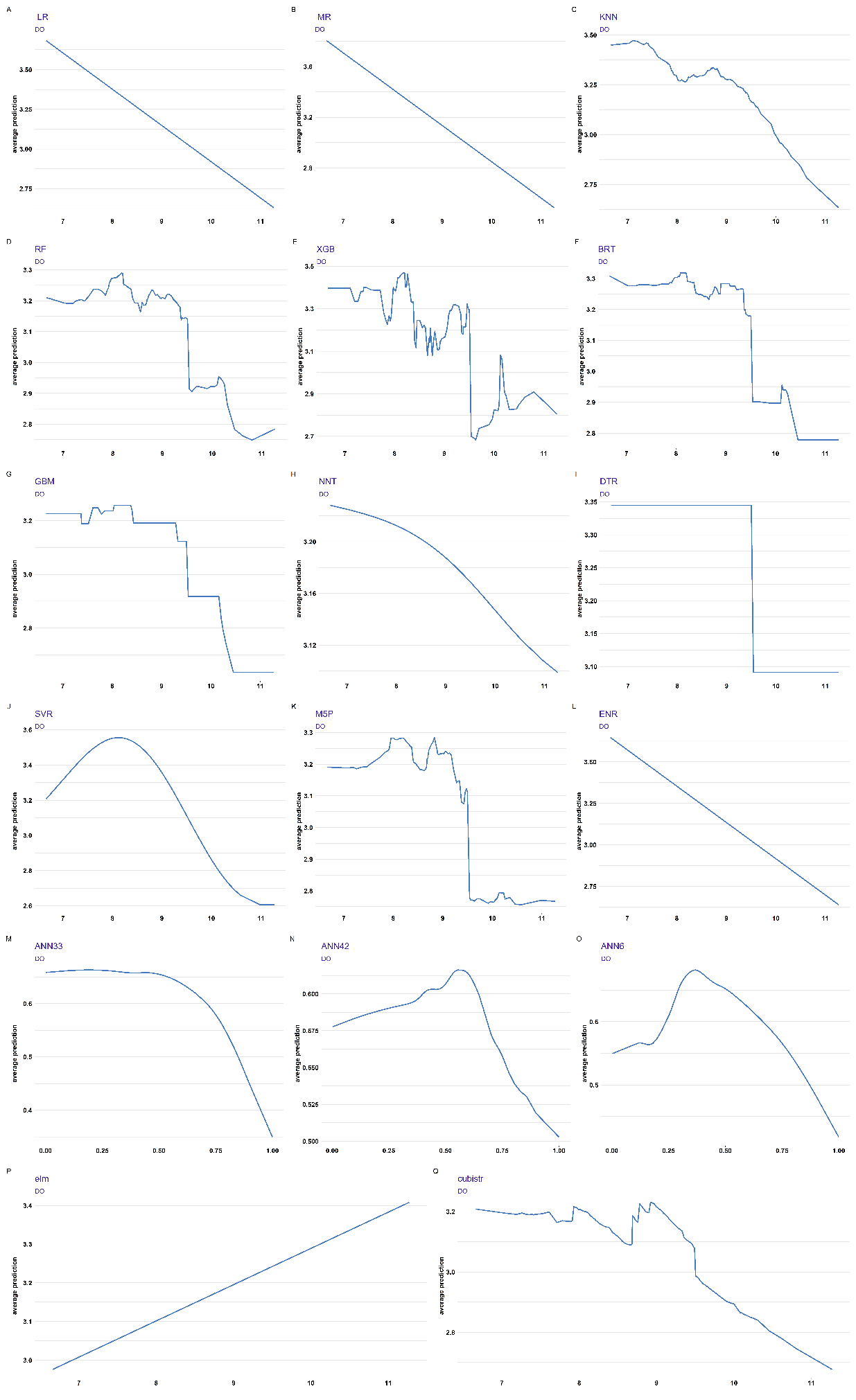


Figure S2: Contrastive partial-dependence profiles of dissolved oxygen in predicting AD by eighteen models.


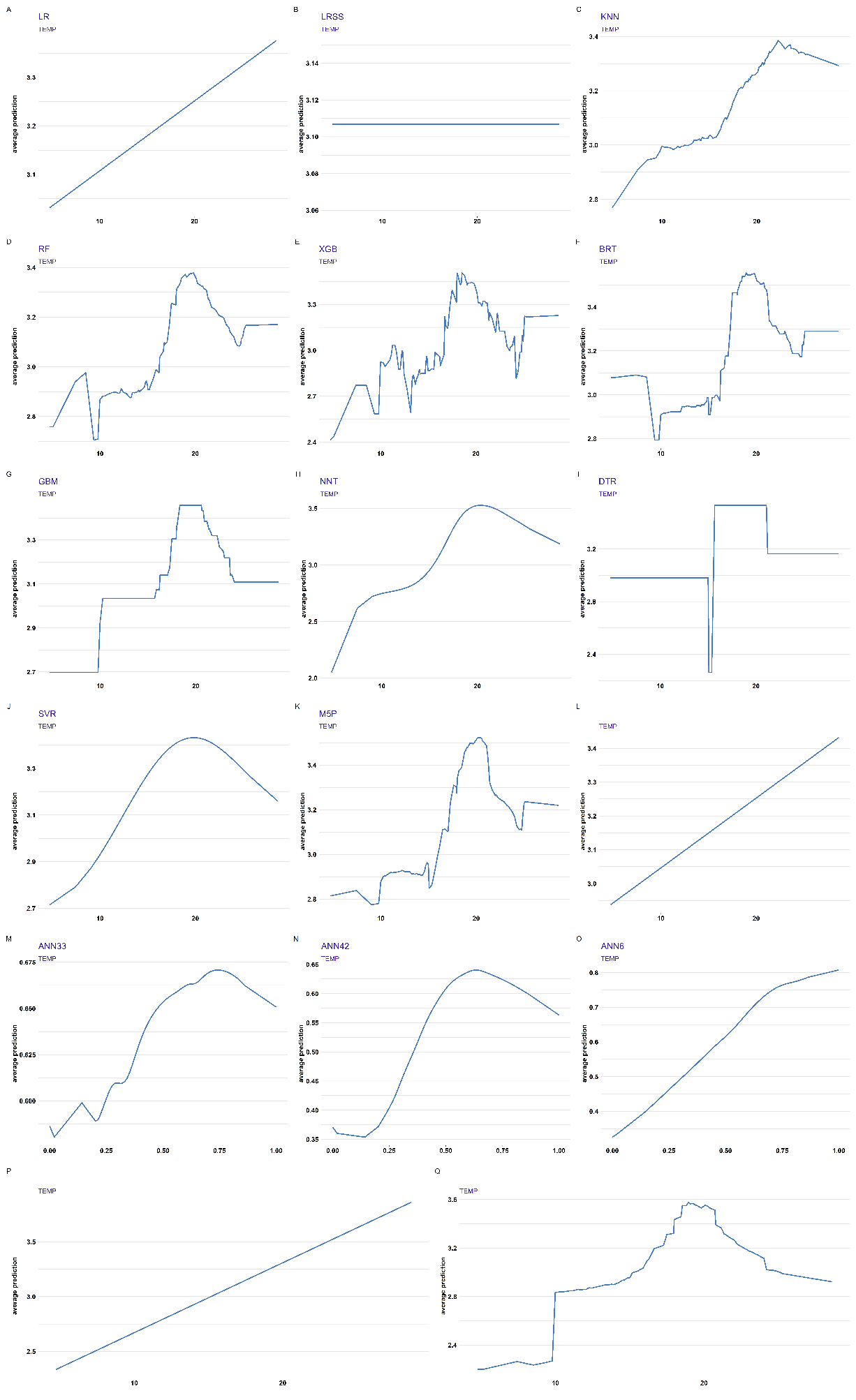


Figure S3: Contrastive partial-dependence profiles of temperature in predicting AD by eighteen models.


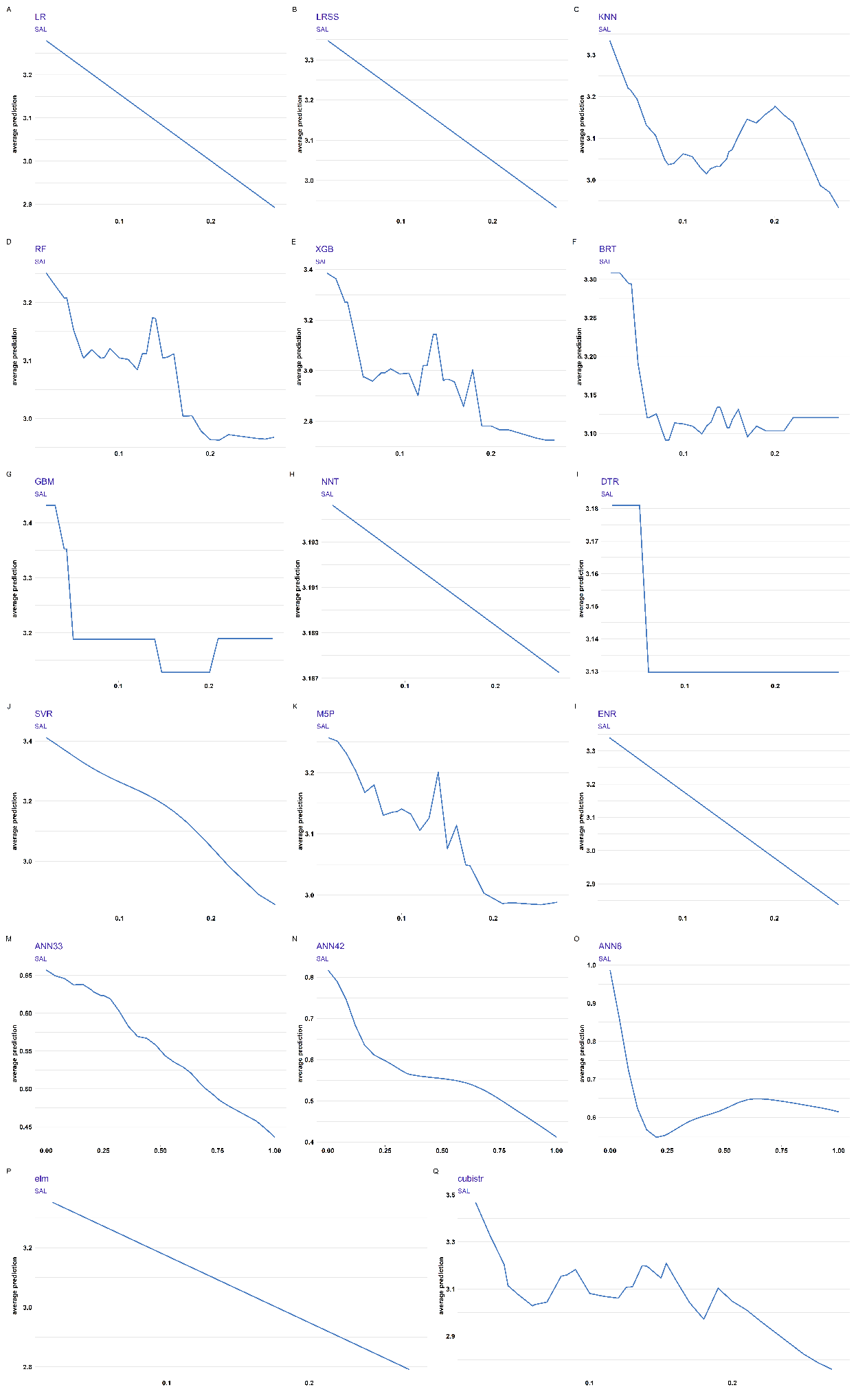


Figure S4: Contrastive partial-dependence profiles of salinity in predicting AD by eighteen models.


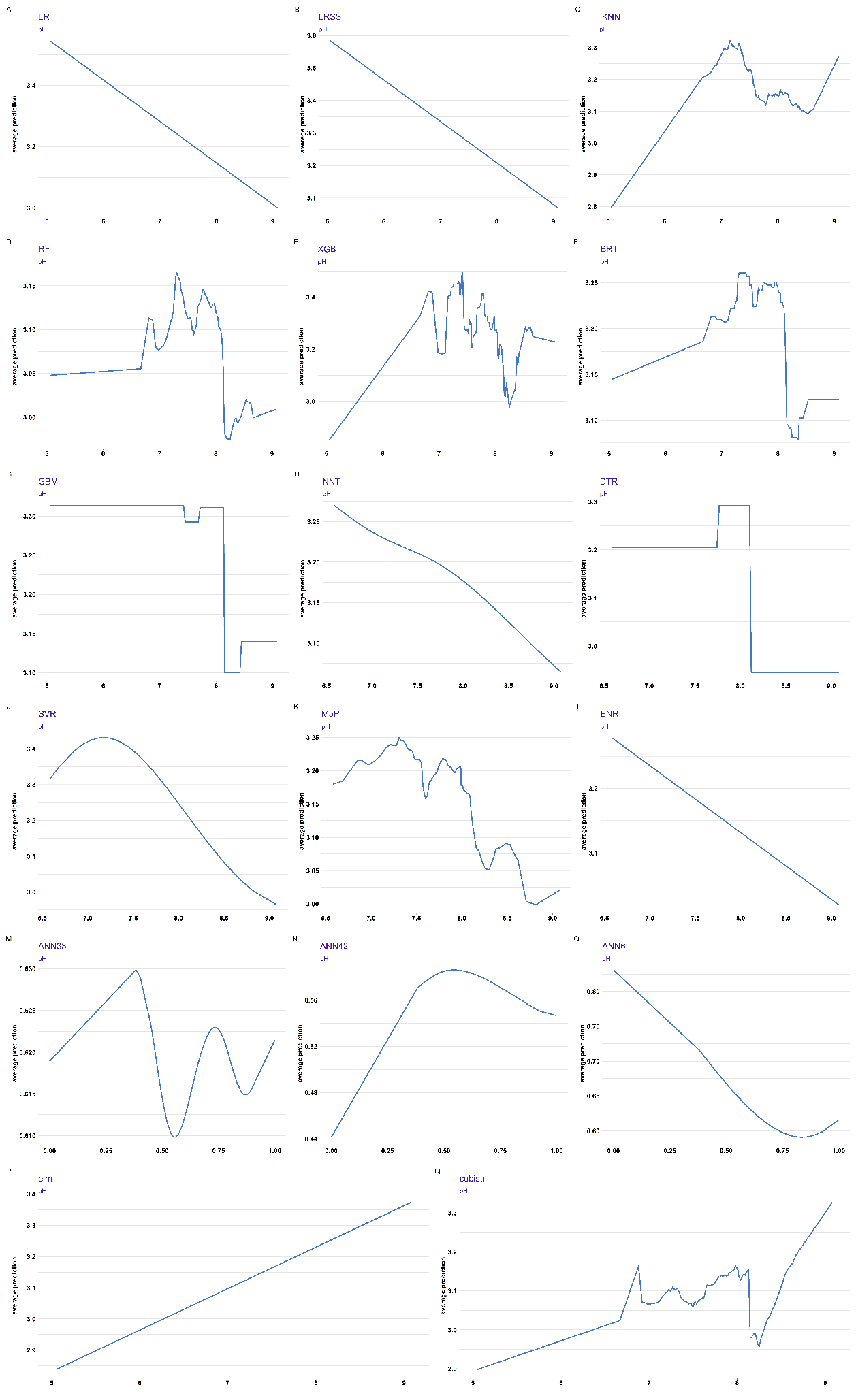


Figure S5: Contrastive partial-dependence profiles of pH in predicting AD by eighteen models.


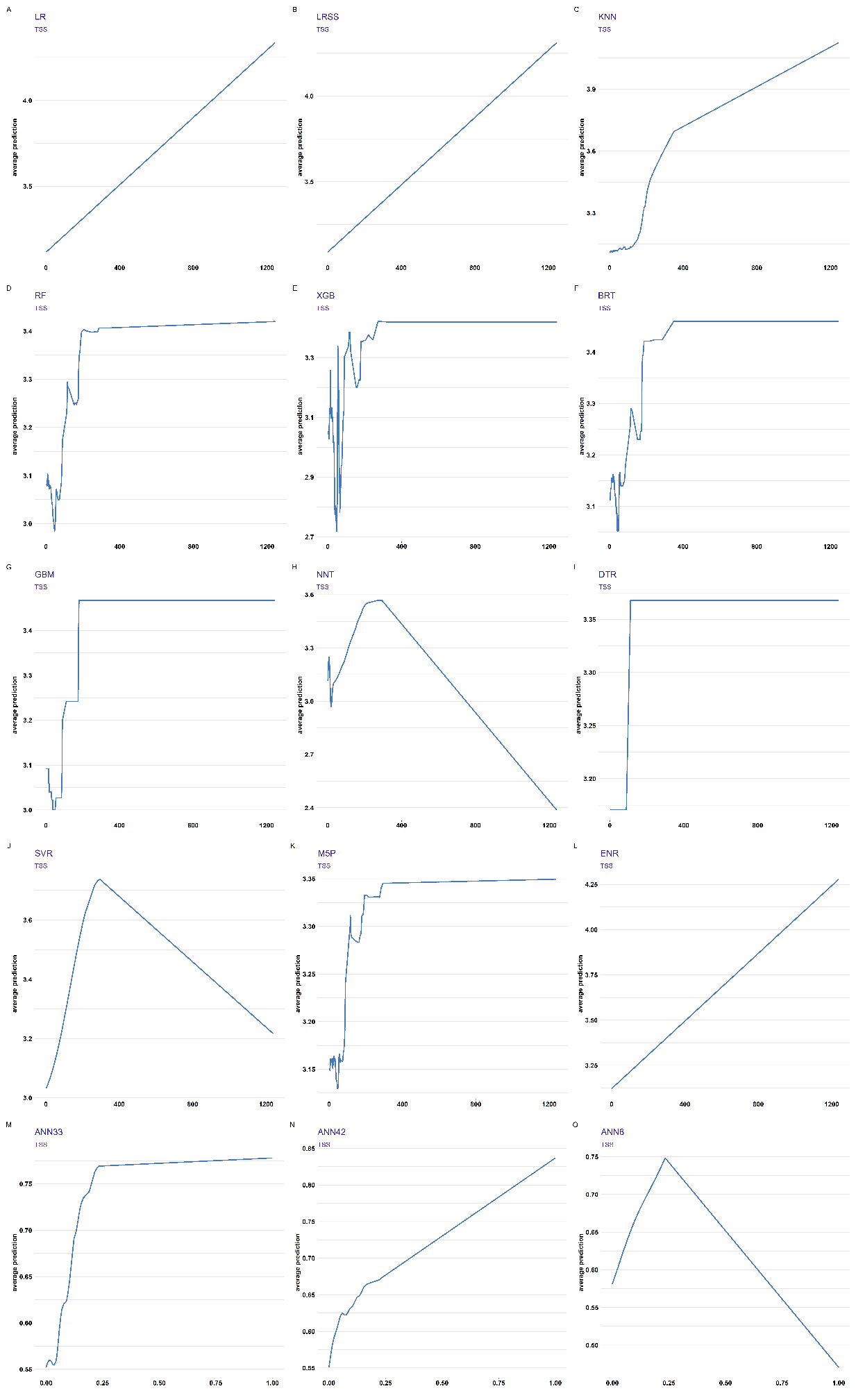


Figure S6: Contrastive partial-dependence profiles of total suspended solids in predicting AD by eighteen models.


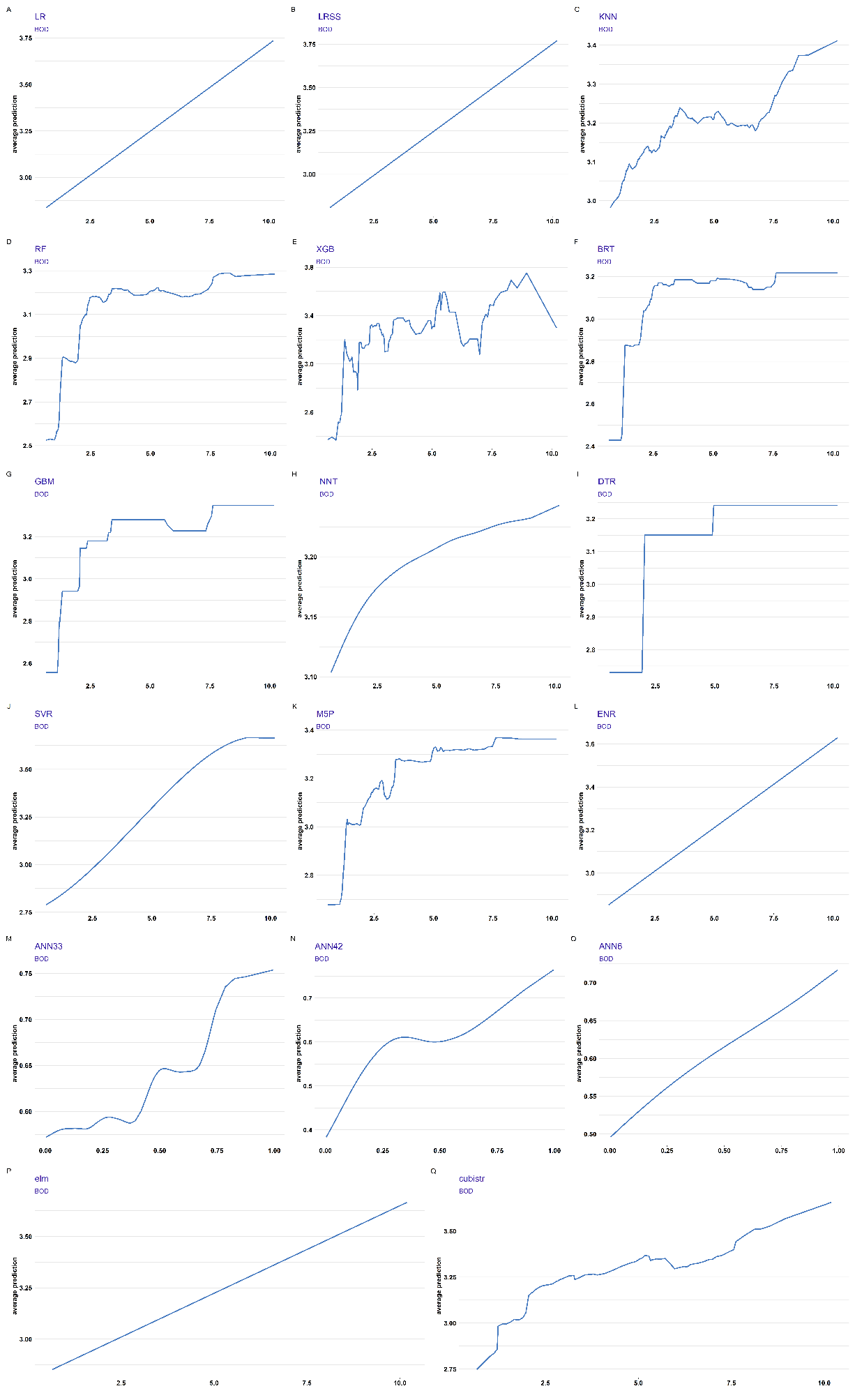


Figure S7: Contrastive partial-dependence profiles of biological oxygen demand in predicting AD by eighteen models.
